# ForestQC: Quality control on genetic variants from next-generation sequencing data using random forest

**DOI:** 10.1371/journal.pcbi.1007556

**Published:** 2019-12-18

**Authors:** Jiajin Li, Brandon Jew, Lingyu Zhan, Sungoo Hwang, Giovanni Coppola, Nelson B. Freimer, Jae Hoon Sul

**Affiliations:** 1 Department of Human Genetics, David Geffen School of Medicine, University of California, Los Angeles, Los Angeles, CA, United States of America; 2 Interdepartmental Program in Bioinformatics, University of California, Los Angeles, Los Angeles, CA, United States of America; 3 Molecular Biology Institute, University of California, Los Angeles, Los Angeles, CA, United States of America; 4 Department of Psychiatry and Biobehavioral Sciences, University of California, Los Angeles, Los Angeles, CA, United States of America; Johns Hopkins University, UNITED STATES

## Abstract

Next-generation sequencing technology (NGS) enables the discovery of nearly all genetic variants present in a genome. A subset of these variants, however, may have poor sequencing quality due to limitations in NGS or variant callers. In genetic studies that analyze a large number of sequenced individuals, it is critical to detect and remove those variants with poor quality as they may cause spurious findings. In this paper, we present ForestQC, a statistical tool for performing quality control on variants identified from NGS data by combining a traditional filtering approach and a machine learning approach. Our software uses the information on sequencing quality, such as sequencing depth, genotyping quality, and GC contents, to predict whether a particular variant is likely to be false-positive. To evaluate ForestQC, we applied it to two whole-genome sequencing datasets where one dataset consists of related individuals from families while the other consists of unrelated individuals. Results indicate that ForestQC outperforms widely used methods for performing quality control on variants such as VQSR of GATK by considerably improving the quality of variants to be included in the analysis. ForestQC is also very efficient, and hence can be applied to large sequencing datasets. We conclude that combining a machine learning algorithm trained with sequencing quality information and the filtering approach is a practical approach to perform quality control on genetic variants from sequencing data.

## Author summary

Genetic disorders can be caused by many types of genetic mutations, including common and rare single nucleotide variants, structural variants, insertions, and deletions. Nowadays, next-generation sequencing (NGS) technology allows us to identify various genetic variants that are associated with diseases. However, variants detected by NGS might have poor sequencing quality due to biases and errors in sequencing technologies and analysis tools. Therefore, it is critical to remove variants with low quality, which could cause spurious findings in follow-up analyses. Previously, people applied either hard filters or machine learning models for variant quality control (QC), which failed to filter out those variants accurately. Here, we developed a statistical tool, ForestQC, for variant QC by combining a filtering approach and a machine learning approach. We applied ForestQC to one family-based whole-genome sequencing (WGS) dataset and one general case-control WGS dataset, to evaluate it. Results show that ForestQC outperforms widely used methods for variant QC by considerably improving the quality of variants. Also, ForestQC is very efficient and scalable to large-scale sequencing datasets. Our study indicates that combining filtering approaches and machine learning approaches enables effective variant QC.

This is a *PLOS Computational Biology* Software paper.

## Introduction

Over the past few years, genome-wide association studies (GWAS) have been playing an essential role in identifying genetic variations associated with diseases or complex traits [1,2]. GWAS have found many associations between common variants and human diseases, such as schizophrenia [3], type 2 diabetes [4,5], and Parkinson’s Disease [6]. However, these common variants typically explain only a small fraction of heritability for the complex traits [7,8]. Rare variants have been considered as an important risk factor for complex traits and diseases [9–12]. With the next-generation sequencing (NGS) technology, geneticists may now gain insights into the roles of novel or rare variants. For instance, deep targeted sequencing was applied to discover rare variants associated with inflammatory bowel disease [13]. Whole-genome sequencing (WGS) has been used to identify rare variants associated with prostate cancer [14], and with whole-exome sequencing, studies have also detected rare variants associated with LDL cholesterol [15] and autism [16].

However, several factors may adversely influence the quality of variants detected by sequencing. First, NGS is known to have errors or biases [17–21], which might cause inaccuracy in detecting variants. Second, the sequence mappability of different regions may not be uniform but correlated with sequence-specific biological features, leading to alignment biases. For instance, it is shown that introns have significantly lower mappability levels than exons [22]. Third, variant calling algorithms may be the sources of errors as no algorithm is 100% accurate. For example, GATK HaplotypeCaller and GATKUnifiedGenotyper [23], which are the widely used variant callers, have a sensitivity of about 96% and precision of about 98% [24]. Additionally, different variant callers may generate discordant calls [25], and in some instances, different versions of even the same software may generate inconsistent calls. All these factors may generate false-positive variants or incorrect genotypes, which may then lead to false-positive associations in the follow-up association analyses. For example, Alzheimer’s Disease Sequencing Project has reported that they found spurious associations in the case-control analysis where one of the causes for the problem could be inconsistent variant discovery pipelines [26].

It is vital to perform quality control (QC) on genetic variants identified from sequencing to remove variants that may contain sequencing errors and hence, are likely to be false-positive calls. Traditionally, genetic studies have utilized two types of QC approaches; we call them “filtering” and “classification” approaches. In the filtering approach, several filters are applied to remove problematic variants such as variants with high genotype missing rate (e.g. > 5%), low Hardy-Weinberg Equilibrium (HWE) p-value (e.g. < 1E-4), or very high or low allele balance of heterozygous calls (ABHet) (e.g. > 0.75 or < 0.25). One main problem with this type of approach is that these thresholds are often study-specific and need to be manually fine-tuned for each study. We may also remove variants whose metrics are very close to the thresholds (e.g., variants with a missing rate of 5.1%). Another type of QC is the classification approach that attempts to learn variants with low quality using machine learning techniques. One example is Variant Quality Score Recalibration (VQSR) of GATK [24,27] that uses a Gaussian mixture model to learn the multidimensional annotation profile of variants with high and low quality. However, one of the issues with VQSR is that one needs training datasets acquired from existing databases on variants such as 1000 Genomes Project [28] and HapMap [29], which may be biased to keep known variants and filter out novel variants. Another issue is that those known databases of genetic variants may not always be accurate, which would lead to inaccurate classification of variants, and they may not even be available for some species. It may also be a challenge to apply VQSR to a variant call set generated by variant callers other than GATK as VQSR needs metrics of variants that are not often calculated by non-GATK variant callers.

In this article, we present ForestQC for performing QC on genetic variants discovered through sequencing. Our method aims to identify whether a variant is of high sequencing quality (high-quality variants) or low quality (low-quality variants) by combining the filtering and classification approaches. We first apply the filtering approach by applying stringent filters to identify truly high-quality or low-quality, while the rest of variants that are neither high-quality nor low-quality are considered to have uncertain quality (“undetermined” variants). Given this set of high-quality and low-quality variants, we train a machine learning model whose goal is to classify whether the undetermined variants are high-quality or low-quality. With an insight that high-quality variants would have higher genotype quality and sequencing depth than do low-quality variants, we use the information of several sequencing quality measures of variants for model training. ForestQC then uses sequencing quality measures of the undetermined variants to predict whether each undetermined variant has high or low sequencing quality. Our approach is different from the filtering strategy in that it only uses filters to identify unambiguously high-quality and low-quality variants and does not attempt to classify undetermined variants with filters. Our method is also different from VQSR as our training strategy allows us to train our model without reference datasets for variants and solves several issues with VQSR mentioned above. Another advantage of our software is that it can be applied to Variant Call Format (VCF) files from most of variant callers that generate standard quality information for genotypes and is very efficient.

To demonstrate the accuracy of ForestQC, we apply it to two high-coverage WGS datasets; 1) large extended pedigrees ascertained for bipolar disorder (BP) from Costa Rica and Colombia [30], and 2) a sequencing study for Progressive Supranuclear Palsy (PSP). The first dataset includes 449 related individuals from families, while the latter dataset consists of 495 unrelated individuals. We show that ForestQC outperforms VQSR and a filtering approach based on ABHet as high-quality variants detected from ForestQC have higher sequencing quality than those from VQSR and the filtering approach in both datasets. This suggests that our tool identifies high-quality variants with higher accuracy than other approaches in both family and unrelated datasets. ForestQC is publicly available at https://github.com/avallonking/ForestQC.

## Results

### Overview of ForestQC

**Fig 1:**
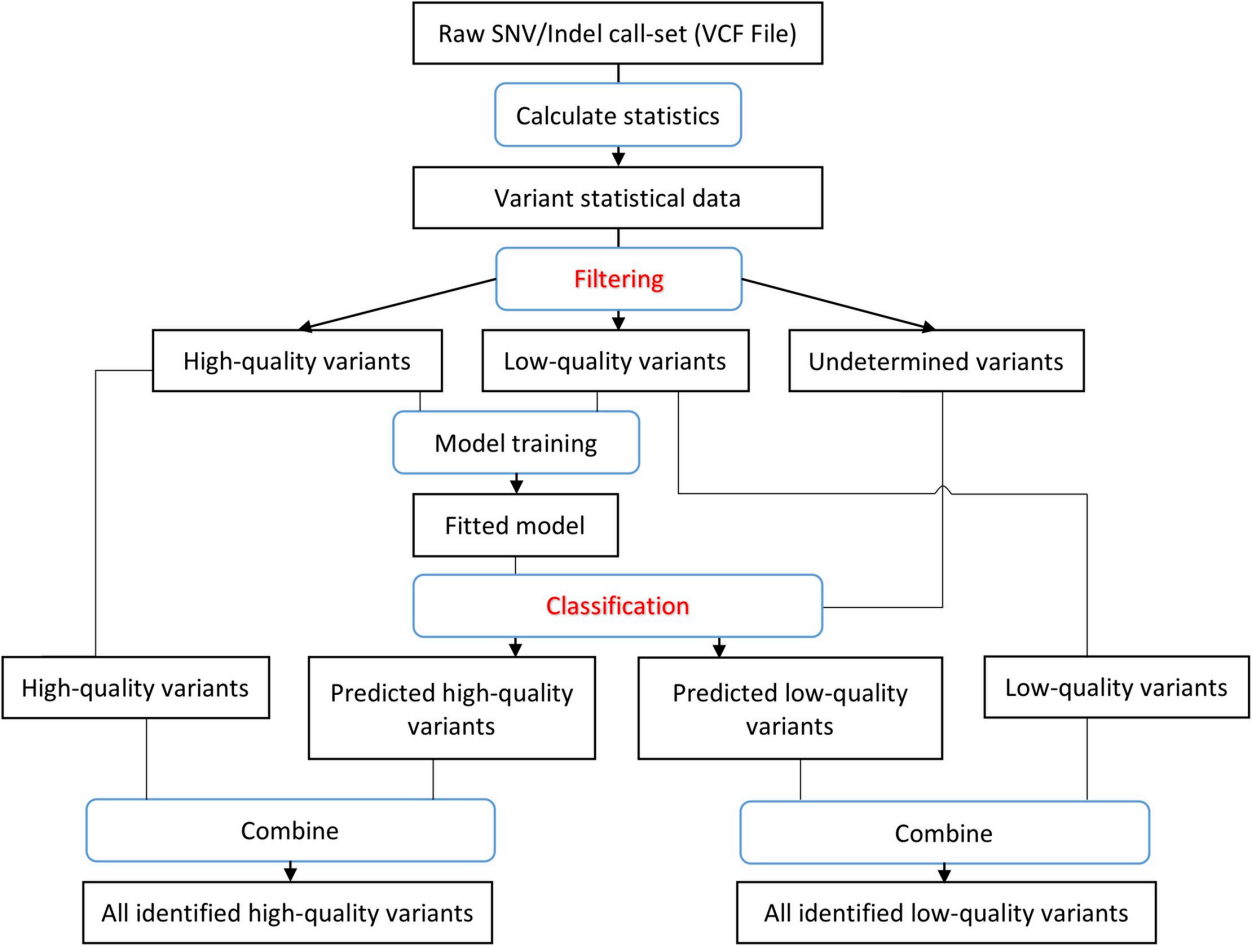
Workflow of ForestQC. ForestQC takes a raw variant call set in the VCF format as input. Then it calculates the statistics of each variant, including MAF, mean depth, mean genotyping quality. In the filtering step, it separates the variant call set into high-quality, low-quality, and undetermined variants by applying various hard filters, such as Mendelian error rate and genotype missing rate. In the classification step, high-quality and low-quality variants are used to train a random forest model, which is then applied to assign labels to undetermined variants. Variants predicted to be high-quality among undetermined variants are combined with high-quality variants from the classification step for the final set of high-quality variants. The same procedure applies to find the final set of low-quality variants.

ForestQC takes a raw VCF file as input and determines which variants have high or low quality. Our method combines a filtering approach that determines high-quality and low-quality variants by a set of pre-defined filters and a classification approach that uses machine learning to classify whether a variant is high-quality or low-quality. As illustrated in Fig 1, our method first calculates the statistics of each variant for several filters that are commonly used in performing QC in GWAS. These statistics consist of ABHet, HWE p-value, genotype missing rate, Mendelian error rate for family-based datasets, and any user-defined statistics (details described in Materials and Methods). ForestQC then identifies three sets of variants using these statistics as filters: 1) a set of high-quality variants that pass all filters, 2) a set of low-quality variants that fail any filter(s), and 3) a set of undetermined variants that are neither high-quality nor low-quality variants. We use stringent thresholds for filters (S1 and S2 Tables), and hence we are highly confident that high-quality variants have good quality while low-quality variants are indeed false-positives or have unequivocally poor sequencing quality. The next step in ForestQC is to train a random forest machine learning model using the high-quality and low-quality variants we detect in the filtering step. In ForestQC, seven sequencing quality metrics of high-quality and low-quality variants are used as features to train the random forest model, including three related to sequencing depth, three related to genotype quality, and one related to the GC content. Finally, the fitted model predicts whether each undetermined variant is high-quality or low-quality. We combine the predicted high-quality variants from the random forest classifier and the high-quality variants detected in the filtering step, as the complete set of high-quality variants determined by ForestQC. The same procedure is applied to identify low-quality variants.

One major challenge in classifying undetermined variants is to identify a set of sequencing quality metrics that are used as features to train the random forest model. We choose three sets of features based on quality metrics provided by variant callers and prior knowledge in genome sequencing. The first set of features is genotype quality (GQ), where we have three metrics: mean, standard deviation (SD), and outlier ratio. The outlier ratio is the proportion of samples whose GQ scores are lower than a particular threshold, and it measures a fraction of individuals who are poorly sequenced at a mutation site. A high-quality variant is likely to have high mean, low SD, and low outlier ratio of GQ values. The second set of features is sequencing depth (DP), as low depth often introduces sequencing biases and reduces variant calling sensitivity [31]. We also use the same three sets of metrics for DP as those for GQ: mean, SD, and outlier ratio. The last set of features is related to genomic characteristics instead of sequencing quality, which is GC content. Too high or too low GC content may decrease the coverage of certain regions [32,33] and thus may lower the quality of variant calling. Hence, the GC content of the DNA region containing high-quality variants would not be too high or too low. Given these three sets of features, ForestQC learns how those features determine high-quality and low-quality variants, and classifies undetermined variants according to the rules that it learns.

### Comparison of different machine learning algorithms

As there are various machine learning algorithms available, we first seek to find the most accurate and efficient algorithm for performing QC on NGS variant call-sets. To ensure the quality of training and prediction, we choose supervised learning algorithms rather than unsupervised algorithms. Several major types of supervised algorithms are selected for comparison: random forest, logistic regression, k nearest neighbors (KNN), Naive Bayes, quadratic discriminant analysis (QDA), AdaBoost, artificial neural network (ANN), and single support vector machine (SVM). We use the BP WGS dataset, which consists of large pedigrees from Costa Rica and Colombia, to compare the performance of different algorithms. We use the three sets of features mentioned above for all these algorithms. We apply the filtering approach (S1 and S2 Tables) to the BP data to identify high-quality, low-quality, and undetermined variants, and we randomly sample 100,000 high-quality and 100,000 low-quality variants for model training. We then randomly choose another 100,000 high-quality and 100,000 low-quality variants from the rest of variants for model testing. Each learning algorithm will be trained with the same training set and tested with the same test set. We use 10-fold cross-validation and calculate area under the receiver operating characteristic curve (AUC) and F1-score to estimate classification accuracy during model testing. F1-score is the harmonic average of precision (positive predictive value) and recall (sensitivity). The closer the F1-score is to 1, the better the performance is. To assess the efficiency of each algorithm, we measure its runtime during training and predicting. We use eight threads for algorithms that support parallelization.

Results show that random forest is the most accurate model in both SNV classification and indel classification with the highest F1-scores, accuracy, and the largest AUC (Table 1, S3 Table, S1 Fig). Its runtime is only 9.85 seconds in model training and prediction (Table 1), which ranks as the fourth fastest algorithm. As random forest randomly divides the entire dataset into several subsets of the same size and constructs decision trees independently in each subset, it is highly scalable, and it has low error rates and high robustness [34]. As for other machine learning algorithms, both SVM and ANN are highly accurate (both with F1-score of 0.97 and AUC > 0.985 in SNV classification), but they are not as efficient as random forest. ANN is the second slowest algorithm that is about 8x slower than random forest because it estimates many parameters. Especially, SVM is the slowest algorithm because of its inability to parallelize, which needs about 125x as much time as random forest (Table 1). This suggests that it may be computationally costly to use SVM in large-scale WGS datasets that have tens of millions of variants. Typically, a real dataset is at least ten times larger than the dataset used here. For example, in the BP dataset, the training set has 2.20 million (M) SNVs, and there are 2.73M undetermined SNVs for prediction. We find that random forest only spends 80.51 seconds in training and predicting, while ANN needs 489.63 seconds, and SVM needs 14.74 hours. Therefore, random forest is much faster than ANN and SVM, although all three algorithms have similar performance in terms of AUC (S1 Fig). Also, there are even a more significant number of variants in large-scale WGS projects such as the NHLBI Trans-Omics for Precision Medicine (TOPMed) dataset that includes about 463M variants. Hence, it is more practical to use random forest when processing these massive datasets. Logistic regression, Naive Bayes, and QDA are more efficient than random forest, but their predictions are not as accurate as those of random forest. For example, Naive Bayes needs only 0.18 seconds for training and prediction, while its F1-score is the lowest among all algorithms (0.90 and 0.87 in SNV and indel classification, respectively) (Table 1). This result demonstrates that random forest is both accurate and efficient, and hence we use it as the machine learning algorithm in our approach. To further improve the random forest algorithm, we test a different number of trees in the algorithm, and we find that random forest with 50 trees balances efficiency and accuracy (S2 Fig). Also, we consider undetermined variants with the predicted probability of being high-quality variants > 50% as high-quality variants as this probability threshold achieves the highest F1-score (S3 Fig).

**Table 1:** Performance of eight different machine learning algorithms.

Machine learning algorithm	Time cost (sec)	F1-score for indel classification	F1-score for SNV classification
Random Forest	9.85	0.9428	0.9740
ANN	75.34	0.9400	0.9707
SVM	1253.48	0.9381	0.9704
AdaBoost	25.27	0.9270	0.9672
Logistic Regression	2.49	0.9074	0.9668
KNN	24.71	0.9200	0.9486
QDA	0.30	0.9006	0.9241
Naïve Bayes	0.18	0.8716	0.9012

Performance metrics, including F1-scores, total time cost of model fitting and prediction, are ranked by F1-score for SNV classification. Random forest, ANN, logistic regression and KNN are set to run with eight threads. “ANN”: artificial neural network. “SVM”: single support vector machine. “KNN”: K-nearest neighbors classifier. “QDA”: quadratic discriminant analysis.

### Measuring performance of QC methods on WGS data

To evaluate the accuracy of ForestQC and other methods on WGS data, we apply them to two WGS datasets and calculate several metrics. For a family-based dataset, we calculate the Mendelian error rate (ME) of each variant, which measures inconsistency in genotypes between parents and children. Another metric is the genotype discordance rate between microarray and sequencing as individuals in both WGS datasets we analyze are genotyped with both microarray and WGS. These two metrics are important indicators of variant quality because high-quality variants would follow Mendelian inheritance patterns, and their genotypes would be consistent between microarray and sequencing. Additionally, we compute some other metrics that are reported in sequencing studies such as the number of variants (SNVs and indels), transitions/transversions (Ti/Tv) ratio, the number of multi-allelic variants, genotype missing rate. Note that these QC-related metrics are computed separately for SNVs and indels. We use these metrics to compare the performance of ForestQC with that of three approaches. The first is one without performing any QC (no QC). The second method is VQSR, which is a classification approach that requires known truth sets for model training, such as HapMap or 1000 genomes. We use the recommended resources and parameter settings to run VQSR as of 2018-04-04 [35], but we also look at different settings. The third method is the ABHet approach, which is a filtering approach that retains variants according to the allele balance of variants (see Methods).

### Performance of ForestQC on family WGS data

We apply ForestQC to the BP WGS dataset that consists of 449 subjects with an average coverage of 36-fold. There are 25.08M SNVs and 3.98M indels [30]. The variant calling is performed with GATK-HaplotypeCaller v3.5. This is an ideal dataset for assessing the performance of different QC methods because this dataset contains individuals from families who are both sequenced and genotyped with microarray. This study design allows us to calculate both ME rate and genotype discordance rate of variants between WGS and microarray. For this dataset, we test ForestQC with two different filter settings, one using ME rate as a filter and the other not using ME as a filter. The results of the former approach would filter out low-quality variants based on ME rate, and hence ME rate of high-quality variants would be very low. However, we observe that both approaches have similar performance in terms of ME rate and other metrics (S4 Table, S4 Fig, S5 Fig), and hence we show results of only ForestQC using ME rate as a filter.

**Fig 2:**
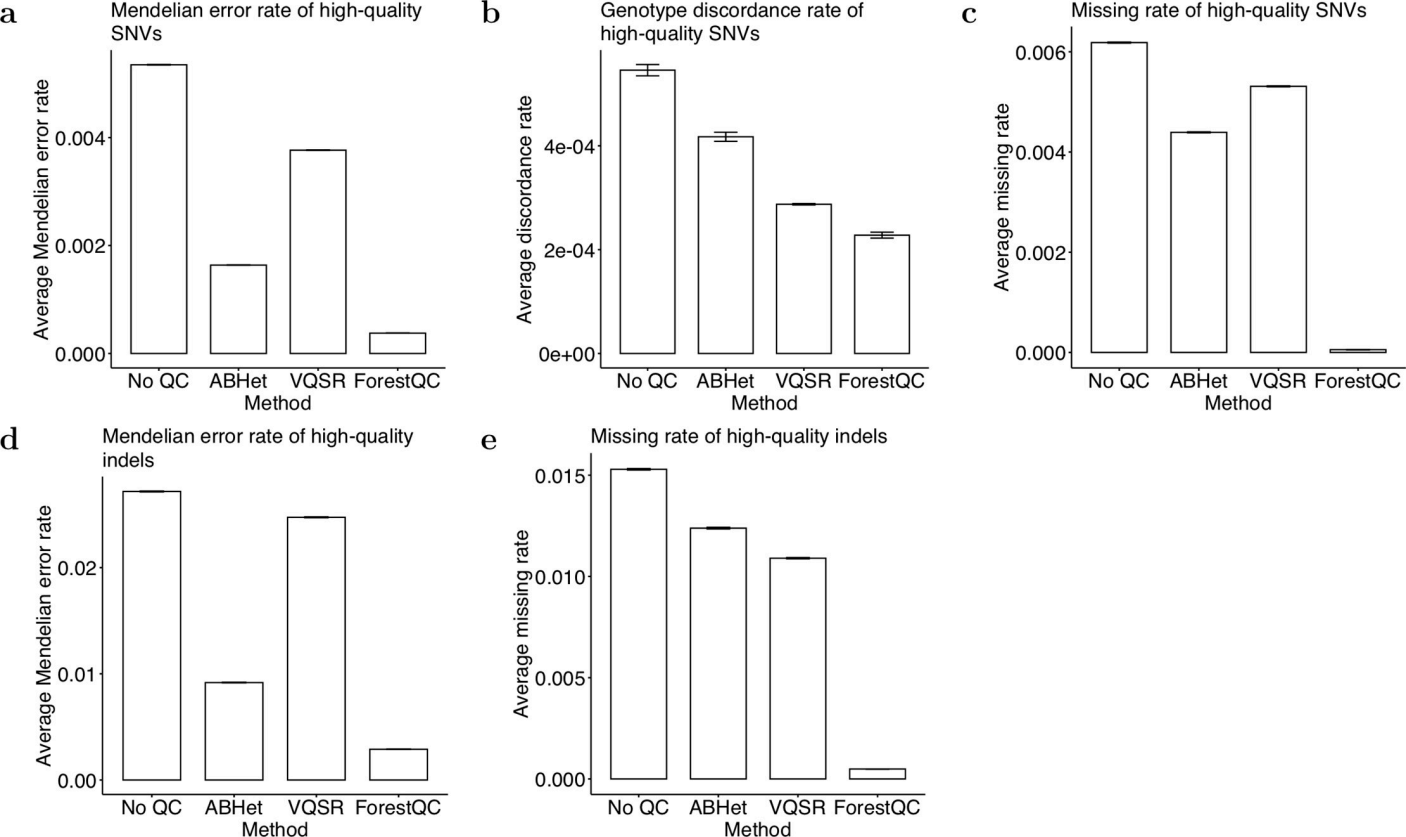
Overall quality of high-quality variants in the BP dataset detected by four different methods. (a) The ME rate, (b) the genotype discordance rate, and (c) the missing rate of high-quality SNVs. (d) The ME rate and (e) the missing rate of high-quality indels. Data are represented as the mean ± SEM.

Results show that ForestQC outperforms ABHet and VQSR in terms of the quality of high-quality SNVs while it detects fewer such SNVs than the other approaches (detailed variant-level metrics in Table S5). ForestQC identifies 22.23M (88% of total SNVs) high-quality SNVs, which is fewer than 22.42M (89%) and 24.24M (97%) high-quality SNVs from ABHet and VQSR, respectively (Table 2). However, ABHet has 3.57x and VQSR has 9.99x higher ME rate on high-quality SNVs than ForestQC (Fig 2A), and ABHet has 1.50x (p-value < 2.2e-16) and VQSR has 1.26x higher genotype discordance rate (p-value < 2.2e-16) on high-quality SNVs than ForestQC (Fig 2B). Besides, ABHet and VQSR have 81.48x and 97.72x higher genotype missing rate on high-quality SNVs than ForestQC, respectively (Fig 2C). However, it is important to note that genotype missing rate is used as a filter in ForestQC, so SNVs with high genotype missing rate are filtered out. We observe that VQSR and ABHet have 319 thousand (K) (1.32% of total high-quality SNVs) and 235K (1.05%) high-quality SNVs with very high genotype missing rate (>10%), respectively, and there are also 118K (0.49%, VQSR) and 53K (0.24%, ABHet) high-quality SNVs with very high ME rate (>15%), while ForestQC has none of them due to its filtering approach. We then investigated whether low-quality variants detected by ForestQC are of poor sequencing quality. Our results show that low-quality SNVs detected by our method have higher genotype missing rate, higher ME rates, and higher genotype discordance rate than those of ABHet, and higher genotype missing rate than those of VQSR (S6A, S6B and S6C Fig). The no QC method keeps the greatest number of SNVs (25.08M), but they have the highest ME rate, genotype missing rate, and genotype discordance rate as expected.

**Table 2:** Variant-level quality metrics of high-quality variants in the BP dataset processed by different methods.

Metric	No QC	ABHet	VQSR	ForestQC
Total SNVs	25081636	22415368	24239357	22227503
Known SNVs	21165051	19665276	20675746	19361635
Known SNVs (%)	84.38%	87.73%	85.30%	87.11%
Total indels	3976710	2670647	3212886	2789037
Known indels	3094271	2188996	2758783	2237002
Known indels (%)	77.81%	81.97%	85.87%	80.21%
Multi-allelic SNVs	153836	26549	128894	77693
Multi-allelic SNVs (%)	0.61%	0.12%	0.53%	0.35%

Four methods are compared, including no QC applied, ABHet approach, VQSR and ForestQC. “Known” stands for variants found in dbSNP. The version of dbSNP is 150.

**Fig 3:**
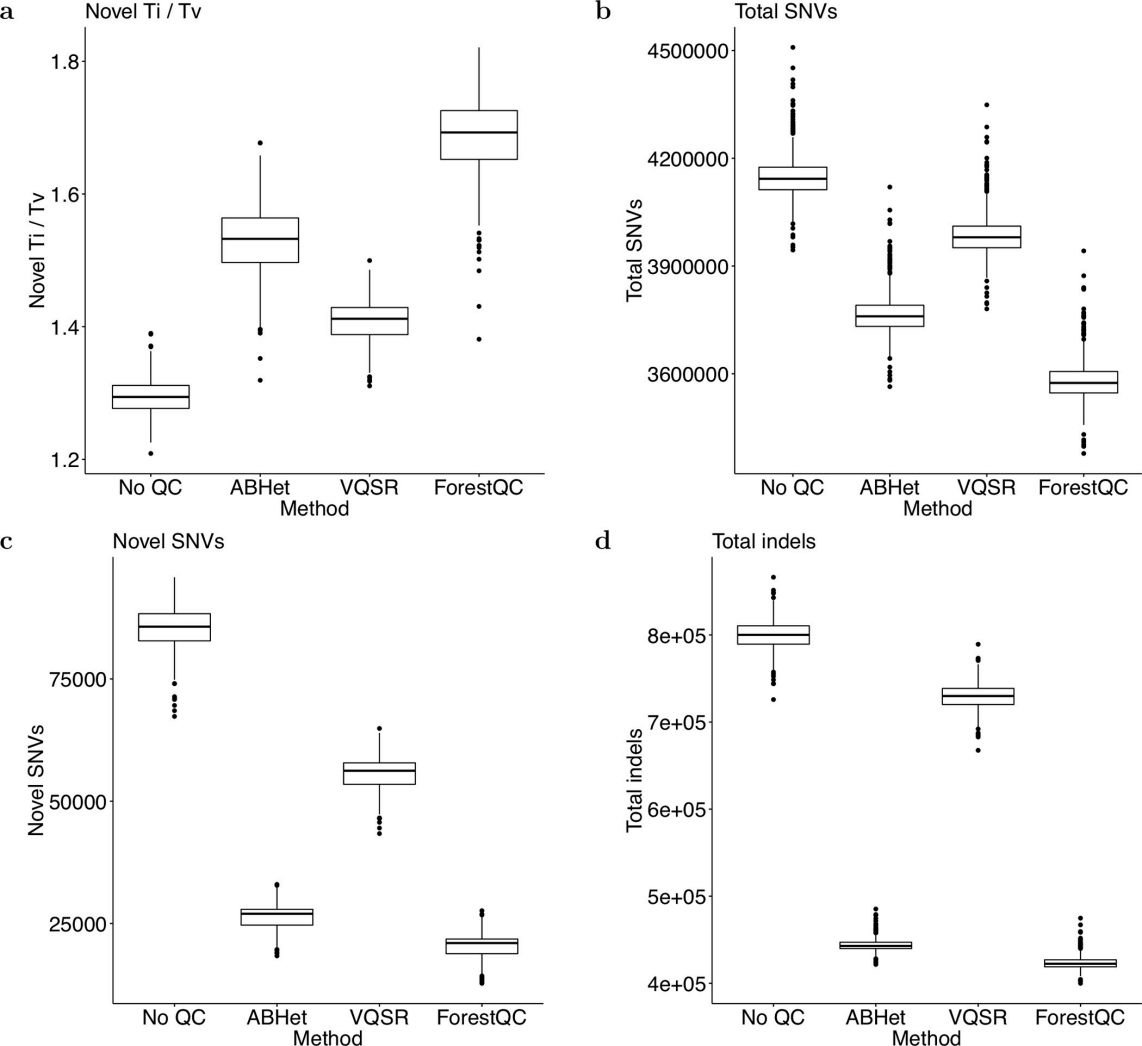
Sample-level quality metrics of high-quality variants in the BP dataset identified by four different methods. (a) Ti/Tv ratio of SNVs not found in dbSNP. (b) The total number of SNVs. (c) The number of SNVs not found in dbSNP. (d) The total number of indels. The version of dbSNP is 150.

Next, we calculate several metrics of high-quality SNVs commonly used in sequencing studies to evaluate the performance of ForestQC. One such metric is the Ti/Tv ratio. It was reported that transitional mutations occurred more frequently than transversional mutations in the human genome [36]. In human WGS datasets, this ratio is expected to be around 2.0 [23]. The lower Ti/Tv ratio is compared to the genome-wide expected value of 2.0, the more false-positive variants are expected in the dataset. We compute the Ti/Tv ratio for each individual across all high-quality SNVs and look at the distribution of those ratios across all individuals (sample-level metrics). We find that the mean Ti/Tv ratio of high-quality known SNVs (present in dbSNP) is around 2.0 for all four methods, which suggests that they have similar accuracy on known SNVs in terms of Ti/Tv ratio (S7A Fig). However, results show that the mean Ti/Tv ratio of high-quality novel SNVs (not in dbSNP) from ForestQC is better than that of those SNVs from other methods; the mean Ti/Tv ratio is 1.68 for ForestQC, which is closest to 2.0 among other methods (1.41 for VQSR, 1.53 for ABHet, and 1.29 for No QC) (Fig 3A). Paired t-tests for the difference in the mean Ti/Tv ratio between ForestQC and other methods are all significant (p-value < 2.2e-16 versus all other methods). This result suggests that novel SNVs predicted to be high-quality by ForestQC are more likely to be true-positives than those novel SNVs from other QC methods. Another metric commonly used in sequencing studies is the percentage of multi-allelic SNVs, which are variants with more than one alternative allele. Given this relatively small sample size (n = 449), true multi-allelic SNVs are not expected to be observed very frequently, so a good portion of them are considered as false-positives. ForestQC has 33.96% and 42.62% smaller fraction of multi-allelic SNVs among high-quality SNVs than do VQSR and no QC methods, while the ABHet approach has the smallest fraction of such SNVs (Table 2). It is important to note that ABHet value is defined as the proportion of reference alleles from heterozygous samples, so ABHet values are not expected to be 0.5 for high-quality multi-allelic mutation sites, but other unknown values. Hence, ABHet does not work properly for multi-allelic variants and may excessively remove multi-allelic SNVs.

In addition to SNVs, we apply the four QC methods to indels. Similar to the results of SNVs, ForestQC identifies fewer high-quality indels than does VQSR, but the quality of those indels from ForestQC is better than that of high-quality indels from ABHet and VQSR. Out of 3.98M indels in total, ForestQC predicts 2.79M indels (70% of total indels) to have good sequencing quality while VQSR and ABHet find 3.21M (81%) and 2.67M (67%) high-quality indels, respectively (Table 2). High-quality indels from VQSR and ABHet, however, have 8.54x and 3.18x higher ME rate, and 22.25x and 25.28x higher genotype missing rate, than those from ForestQC, respectively (Fig 2D and 2E). Low-quality indels identified by ForestQC have 2.25x and 1.32x higher ME rate, and 1.48x and 2.36x higher genotype missing rate than those from VQSR and ABHet, respectively (S6D and S6E Fig). Besides, we observe that there are 95K (2.97% of total high-quality indels, VQSR) and 86K (3.23%, ABHet) high-quality indels with very high genotype missing rate (>10%) and also 167K (5.21%, VQSR) and 44K (1.66%, ABHet) high-quality indels with very high ME rate (>15%), while there are no such indels in ForestQC. This result suggests that many high-quality indels detected by ABHet or VQSR may be false-positives or indels with poor sequencing quality. One of the reasons why VQSR does not perform well on indels could be the reference database it uses for model training as VQSR considers all indels in the reference database (Mills gold standard call set [37] and 1000G Project [38]) as true-positives. This leads VQSR to have a significantly higher proportion of known indels among its high-quality indels (86% of total high-quality indels), compared with 80% from ForestQC and 82% from ABHet (Table 2). Nevertheless, some indels in the reference database may be false-positives or have poor sequencing quality in the variant call-sets of interest. Hence, the performance of VQSR may be limited by using reference database to identify high-quality variants. It is also important to note that in general, indels have much a higher ME rate (0.41% for no QC) than that of SNVs (0.08% for no QC), which is expected given the greater difficulty in calling indels.

Another significant difference between ForestQC and the other approaches is the allele frequency of variants after QC, as ForestQC keeps a higher number of rare variants in its variant set. Our method has 1.77% and 1.64% higher proportion of rare SNVs, and 5.30% and 15.37% higher proportion of rare indels than ABHet and VQSR do, respectively (S6 Table). We also observe this phenomenon in the variant-level and sample-level metrics for the number of SNVs. The variant-level metrics show that the number of high-quality SNVs detected by ForestQC is similar to those from ABHet (Table 2). However, the sample-level metrics show that each individual on average carries fewer alternative alleles of high-quality SNVs from ForestQC (3.58M total SNVs) than those from VQSR and ABHet (3.99M and 3.77M total SNVs, respectively) (Fig 3B and 3C, S7B Fig). We observe a similar phenomenon for indels between ABHet and ForestQC (Table 2, Fig 3D, S7C and S7D Fig). This phenomenon could be explained by the higher fraction of rare variants among high-quality variants from ForestQC, as individuals would carry fewer variants if there are a higher fraction of rare variants. One main reason why ForestQC has a higher proportion of rare variants is that common variants in the BP dataset have higher ME rate, genotype discordance rate, and genotype missing rate than do rare variants, and therefore, they are more likely to fail the filters of ForestQC (S8 Fig).

ForestQC uses several filters to remove low-quality variants while the other two approaches (VQSR and ABHet) do not use these filters, which might have artificially improved the performance of ForestQC. Hence, to compare ForestQC with other approaches without this potential bias, we measure the performance metrics on only undetermined variants as the filters do not determine their quality in our approach. From 2.73M undetermined SNVs and 1.09M undetermined indels, ForestQC identifies 979K (35.83% of total undetermined SNVs) high-quality SNVs and 532K (48.58% of total undetermined indels) high-quality indels, while ABHet approach detects 620K (22.70%) SNVs and 195K (17.80%) indels, and VQSR selects 2.16M (79.18%) SNVs and 643K (58.76%) indels as high-quality variants, respectively (S7 Table). For high-quality SNVs from undetermined variants, ABHet and VQSR have 2.75x and 22.67x higher ME rate than ForestQC, respectively (S9A Fig), and ABHet and VQSR have 5.15x (p-value = 1.367e-14) and 3.86x (p-value = 1.926e-14) higher genotype discordance rate than ForestQC (S9B Fig). Also, ABHet and VQSR have 15.50x and 7.05x higher genotype missing rate on high-quality SNVs than ForestQC, respectively (S9C Fig). We observed similar results for indels (S9D and S8E Figs). Sample-level metrics also show that ForestQC has better Ti/Tv ratio on known SNVs (mean Ti/Tv: 1.64, 1.85, 1.72, 1.88 for No QC, ABHet, VQSR, ForestQC, respectively), and novel SNVs (mean Ti/Tv: 1.14, 1.04, 1.21, 1.22 for No QC, ABHet, VQSR, ForestQC, respectively) than other methods (S10D and S9E Figs). Paired t-tests for the difference in the mean Ti/Tv ratio of novel SNVs and known SNVs between ForestQC and other methods are all significant (p-value < 0.05 versus all other methods). These results show that ForestQC has better performance than ABHet and VQSR, even on those undetermined variants whose quality is not determined by filtering.

### Performance of ForestQC on WGS data with unrelated individuals

To evaluate the performance of ForestQC on WGS datasets that contain only unrelated individuals, we apply it to the PSP dataset that has 495 subjects who are whole-genome sequenced at an average coverage of 29-fold, generating 33.27M SNVs and 5.09M indels. Among the 495 individuals who are sequenced, 381 individuals (77%) of them are also genotyped with microarray, which enables us to check the genotype discordance rate between WGS and microarray data. Because the PSP dataset contains only unrelated individuals, we do not report the ME rate. Similar to the BP WGS dataset, we apply four methods (ForestQC, VQSR, ABHet, and No QC) to the PSP dataset, although the parameter settings of VQSR have slightly changed. As the PSP dataset is called with GATK v3.2, the StrandOddsRatio (SOR) information from the VCF file is missing, which is recommended to use in VQSR. However, we find that SOR information has little impact on the results of VQSR as we test VQSR without SOR information using the BP dataset and obtain similar results with one using SOR information (S11 Fig).

**Fig 4:**
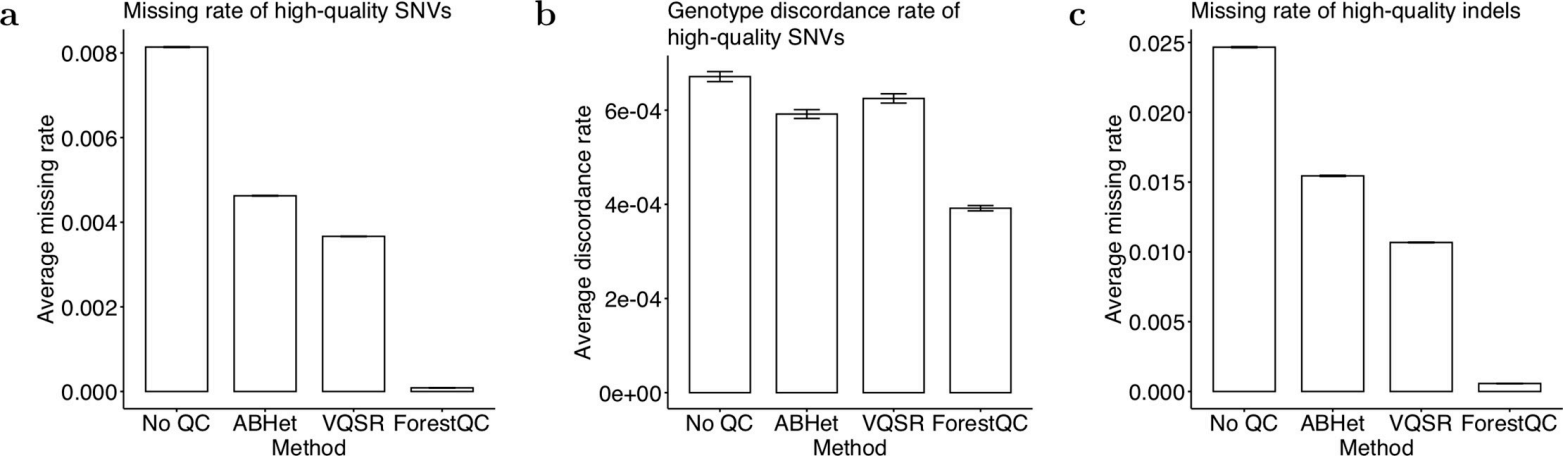
Overall quality of high-quality variants in the PSP dataset detected by four different methods. (a) The missing rate and (b) the genotype discordance rate of high-quality SNVs. (c) The missing rate of high-quality indels. Data are represented as the mean ± SEM.

**Fig 5:**
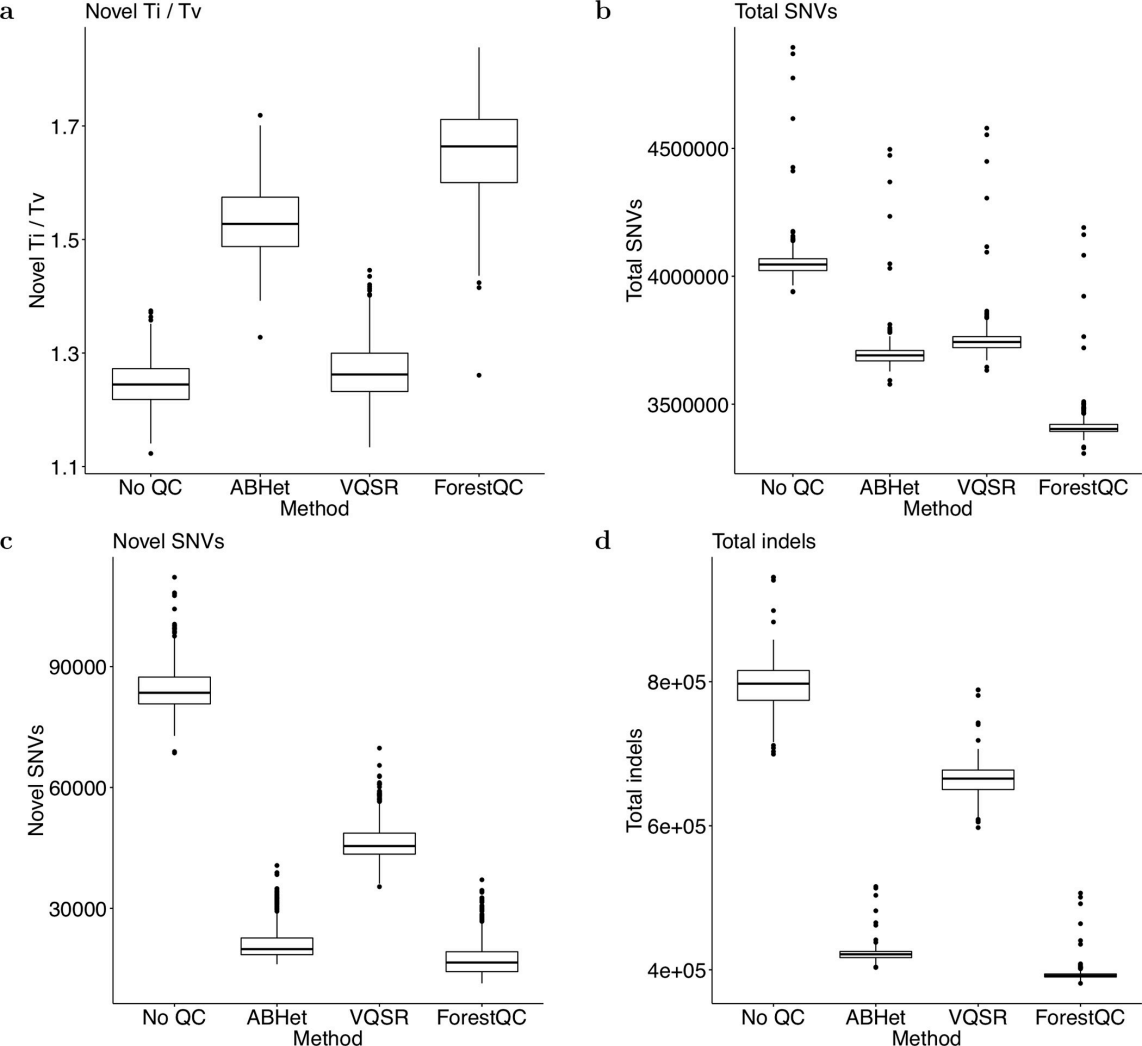
Sample-level quality metrics of high-quality variants in the PSP dataset identified by four different methods. (a) Ti/Tv ratio of SNVs not found in dbSNP. (b) The total number of SNVs. (c) The number of SNVs not found in dbSNP. (d) The total number of indels. The version of dbSNP is 150.

Similar to the results of the BP dataset, high-quality variants identified by ForestQC are fewer but of higher sequencing quality than other approaches (detailed variant-level metrics in Table S8). ForestQC identifies 29.25M (88% of total SNVs) high-quality SNVs, which is slightly fewer than 29.77M (89%) high-quality SNVs from ABHet but about 2 million fewer than 31.28M (94%) high-quality SNVs from VQSR (Table 3). However, high-quality SNVs from ABHet and VQSR have 53.76x and 42.55x higher genotype missing rate than those from ForestQC, respectively (Fig 4A), but it is important to note that missing rate is included as a filter in ForestQC. In addition, there are 311K (0.99% of total high-quality SNVs, VQSR) and 331K (1.13%, ABHet) high-quality SNVs with very high genotype missing rates (>10%), while ForestQC removes all these SNVs. We also observe that low-quality SNVs from ForestQC have a 2.4x higher genotype missing rates than those from ABHet, although low-quality SNVs from GATK have slightly higher missing rates than those from ForestQC (S12A Fig). High-quality SNVs from ABHet and VQSR have 1.28x (p-value < 2.2e-16) and 1.29x higher genotype discordance rate (p-value < 2.2e-16) than those from ForestQC, respectively (Fig 4B). As for the genotype discordance rate of low-quality SNVs, both ABHet and VQSR have higher genotype discordance rate than does ForestQC (S12B Fig), but this may be inaccurate because of the small number of low-quality SNVs genotyped with microarray (10,130, 4,121, and 553 such SNVs for ForestQC, ABHet, and VQSR, respectively). The variant-level and sample-level metrics also indicate the better quality of high-quality SNVs from ForestQC. Although all methods have mean Ti/Tv ratios of high-quality known SNVs above 2.0, the mean Ti/Tv ratio of high-quality novel SNVs among all sequenced individuals is 1.65 for ForestQC, which is closer to 2.0 than other methods (1.27, 1.54, and 1.24 for VQSR, ABHet, no QC, respectively). (S13A Fig, Fig 5A). Paired t-tests for the difference in the mean Ti/Tv ratio between ForestQC and other methods are all significant (p-value < 2.2e-16 versus all other methods). ForestQC has 16.67% and 33.33% smaller fraction of multi-allelic SNVs among high-quality SNVs than do VQSR and no QC methods, respectively, while the ABHet approach has the smallest proportion of such SNVs (Table 3). ABHet has the smallest number of multi-allelic SNVs because of the reason we discussed in the previous BP dataset analysis. Lastly, consistent with the results of the BP dataset, the sample-level metrics show that each individual on average carries fewer alternative alleles of high-quality SNVs from ForestQC than those from VQSR and ABHet (Fig 5B and 5C, S13B Fig). Rare SNVs in high-quality SNVs from ForestQC account for 1.70% and 1.32% higher proportion, compared with those from ABHet and VQSR (S5 Table). This is because rare SNVs in the PSP dataset have lower genotype missing rate and lower genotype discordance rate, and thus do not fail filters as often as do common SNVs (S14A and S14B Fig).

**Table 3:** Variant-level quality metrics of high-quality variants in the PSP dataset processed by four different methods.

Metric	No QC	ABHet	VQSR	ForestQC
Total SNVs	33273111	29771182	31281620	29352329
Known SNVs	25960464	24142744	24910728	23514257
Known SNVs (%)	78.02%	81.09%	79.63%	80.11%
Total indels	5093443	3311136	3682319	3418242
Known indels	3679990	2532899	3012662	2567879
Known indels (%)	72.25%	76.50%	81.81%	75.12%
Multi-allelic SNVs	250418	6685	188180	146247
Multi-allelic SNVs (%)	0.75%	0.02%	0.60%	0.50%

Four methods are compared, including no QC applied, ABHet approach, VQSR and ForestQC. “Known” stands for variants found in dbSNP. The version of dbSNP is 150.

For indels, ForestQC predicts 3.42M indels (67% of total 5.09M indels) to be high-quality variants, which is slightly more than 3.31M (65%) high-quality indels from ABHet and fewer than 3.68M (72%) high-quality indels from VQSR (Table 3). Because the PSP dataset lacks the ME rate as it contains only unrelated individuals and indels are not detected by microarray, it is difficult to compare the performance of the QC methods on indels. We find that high-quality indels from ABHet and VQSR have 27.02x and 18.77x higher genotype missing rate than those from our method, respectively (Fig 4C). Additionally, VQSR and ABHet have 107K (2.91% of total high-quality indels) and 131K (4.08%) high-quality indels with high genotype missing rate (>10%), respectively, while ForestQC filters out all of these indels. Also, low-quality indels from ForestQC have 2.05x and 1.21x higher genotype missing rate than those from ABHet and VQSR, respectively (S12C Fig). This, however, may be biased comparison as ForestQC removes indels with high genotype missing rate in its filtering step. Consistent with the results of SNVs, the sample-level metrics indicate that each individual has fewer high-quality indels from ForestQC than those from VQSR and ABHet (Fig 5D, S13C, S13D Fig). Among high-quality indels, ForestQC has 6% and 1% more novel indels than VQSR and ABHet, respectively (Table 3). In terms of allele frequency, rare indels detected by ForestQC accounts for 12.35% and 3.49% larger proportions than those identified by VQSR and ABHet, respectively (S9 Table). Similar to the results of the BP dataset, we also observe that the missing rate of rare indels is lower than that of common indels. (S14C Fig).

Similar to the analysis of the BP dataset, we also compare the performance of ForestQC, ABHet approach, and VQSR only on undetermined variants in the PSP dataset. From 3.95M undetermined SNVs and 1.60M undetermined indels, ForestQC identifies 1.71M (43.33% of total undetermined SNVs) high-quality SNVs and 719K (45.01% of total undetermined indels) high-quality indels, while ABHet approach detects 780K (19.74%) SNVs and 248K (15.51%) indels, and VQSR selects 2.75M (69.52%) SNVs and 820K (51.34%) indels as high-quality variants, respectively (S10 Table). For high-quality SNVs from undetermined variants, ABHet and VQSR have 14.84x and 5.38x higher genotype missing rate than ForestQC, respectively (S15A Fig). In addition, ABHet has 2.09x (p-value = 2.183e-11) and VQSR has 2.13x higher genotype discordance rate (p-value = 1.584e-10) on than ForestQC (S15B Fig). For indels, ABHet and VQSR have 9.39x and 3.61x higher genotype missing rate on high-quality indels than ForestQC, respectively (S15C Fig). Sample-level metrics also show that ForestQC has better Ti/Tv ratio on known SNVs (mean Ti/Tv: 1.75, 1.87, 1.82, 1.96 for No QC, ABHet, VQSR and ForestQC, respectively) and novel SNVs (mean Ti/Tv: 1.17, 1.03, 1.20, 1.39 for No QC, ABHet, VQSR and ForestQC, respectively) than other methods (S15D and S15E Fig). Paired t-tests for the difference in the mean Ti/Tv ratio of novel SNVs and known SNVs between ForestQC and other methods are all significant (p-value < 2.2e-16 versus all other methods). Similar to the results of the BP dataset, ForestQC has higher accuracy in identifying high-quality variants from undetermined variants, compared with the ABHet approach and VQSR.

### Feature importance in random forest classifier

ForestQC uses several sequencing features in the random forest classifier to predict whether a variant with undermined quality is high-quality or low-quality. To understand how these features determine variant quality, we analyze the feature importance of the fitted random forest classifier. We first find that GC-content has the lowest importance in both BP and PSP datasets and also for both SNVs and indels (S17 Fig). This means that GC-content may not be an informative indicator of the quality of variants as other features related to sequencing quality, such as depth (DP) and genotype quality (GQ). Second, the results show that classification results are not determined by one or two most important features as there is no feature with much higher importance than other features except GC-content. This suggests that all sequencing features except GC-content are essential indicators of the quality of variants and need to be included in our model. We also check correlation among features and find that while specific pairs of features are highly correlated, like outlier GQ and mean GQ, SD DP and mean DP, some features have low correlation to other features, such as GC, suggesting that they may capture different information on quality of genetic variants (S19 Fig). Third, we observe that the same features have different importance between the BP dataset and the PSP dataset. For example, for SNVs, an outlier ratio of the GQ feature has the highest importance for the PSP dataset, while it has the third-lowest importance for the BP dataset (S17A Fig). Also, the importance of features varies between SNVs and indels. For example, SD DP has the highest importance for SNVs in the BP dataset, but it has the third-lowest importance for indels (S17A and S17B Fig). Therefore, these results suggest that each feature may have a different contribution to classification results depending on sequencing datasets and types of genetic variants.

### Performance of VQSR with different settings

For SNVs, GATK recommends three SNV call sets for training its VQSR model; 1) SNVs found in HapMap (“HapMap”), 2) SNVs in the omni genotyping array (“Omni”), and 3) SNVs in the 1000 Genomes Project (“1000G”). According to the VQSR parameter recommendation, SNVs in HapMap and Omni call sets are considered to contain only true variants, while SNVs in 1000G consist of both true- and false-positive variants [35]. We call this recommended parameter setting, “original VQSR.” We, however, find that considering SNVs in Omni to contain both true- and false-positive variants considerably improves the quality of SNVs from VQSR for the BP dataset. We call this modified parameter setting, “Omni_Modified VQSR”. Results show that the mean Ti/Tv on high-quality novel SNVs from Omni_Modified VQSR is 1.76, which is much higher than that from the original VQSR (1.41) and slightly higher than that from ForestQC (1.68) (S19A Fig). We also find that the mean number of total SNVs from Omni_Modified VQSR is 3.68M, which is much smaller than that from the original VQSR (3.99M) but higher than that from ForestQC (3.58M) (S19B Fig). In terms of other metrics, high-quality SNVs from original VQSR have a 3.66x higher ME rate, 7.40x higher genotype missing rate, and 1.16x higher genotype discordance rate (p-value = 0.0001118) than those SNVs from Omni_Modified VQSR (S19C–S19E Fig). Interestingly, we do not observe the improved performance of Omni_Modified VQSR in the PSP dataset as the mean Ti/Tv of high-quality novel SNVs from Omni_Modified VQSR is 1.23, which is slightly smaller than that of original VQSR (1.27) (S19A Fig). Nevertheless, individuals have fewer high-quality SNVs from Omni_Modified VQSR (3.53M) than that from original VQSR (3.75M) (S19B Fig). These results suggest that the performance of VQSR may change significantly depending on whether to consider a reference SNV call-set to contain only true-positive variants or both true- and false-positive variants, and it appears that the difference in performance is more noticeable in certain sequencing datasets than others.

Although Omni_Modified VQSR has slightly better Ti/Tv on high-quality novel SNVs and identifies more high-quality SNVs than does ForestQC, high-quality SNVs from Omni_Modified VQSR have 2.76x higher ME rate, 13.20x higher genotype missing rate, and 1.35x higher genotype discordance rate (p-value < 2.2e-16) than high-quality SNVs from ForestQC (S19C–S19E Fig). Hence, the results show that high-quality SNVs from ForestQC have higher quality than those from VQSR, even with the modified parameter setting.

## Discussion

We developed an accurate and efficient method called ForestQC to identify a set of variants with high sequencing quality from NGS data. ForestQC combines the traditional filtering approach for performing QC in GWAS and the classification approach that uses a machine learning algorithm to classify whether a variant has good quality. ForestQC first uses stringent filters to identify high-quality and low-quality variants that unequivocally have high and low sequencing quality, respectively. ForestQC then trains a random forest classifier using the high-quality and low-quality variants obtained from the filtering step, and predicts whether a variant with ambiguous quality (an undetermined variant) is high-quality or low-quality in an unbiased manner. To evaluate ForestQC, we applied our method to two WGS datasets where one dataset consists of related individuals from families, while the other dataset has unrelated individuals. We demonstrated that high-quality variants identified from ForestQC in both datasets had higher quality than those from other approaches such as VQSR and a filtering approach based on ABHet.

To measure the performance of variant QC methods, one may apply these methods to benchmarking datasets where the true variants with high sequencing quality are verified. A few high-quality benchmarking variant sets have been released, including Genome In A Bottle (GIAB) [39], Platinum Genome (PlatGen) [40], and Syndip [41]. GIAB has seven samples, PlatGen sequenced 17 individuals and derived variant truth sets for two subjects, and Syndip includes only two cell lines, CHM1 and CHM13. The sample sizes of these datasets are very small, while we usually need to perform variant QC on an entire large dataset containing tens of millions of variants from hundreds of subjects or more. In order to apply ForestQC, the variant call-sets should have at least five subjects to calculate the statistics like SD DP and SD GQ accurately. Besides, it is recommended to apply VQSR to variant call-sets with more than 30 samples to achieve reliable results [35]. Thus, these datasets cannot be used as benchmarking datasets for variant QC. Apart, it is not expected to have a new benchmarking dataset with a large sample size soon because it is expensive to construct such a dataset. Hence, in this study, we used real WGS datasets to evaluate different approaches for variant QC. Their large sample sizes allow more accurate calculation of various quality metrics and statistics used by the variant QC methods, and therefore enable more reliable performance evaluation.

To measure the quality of variants, we used 21 sample-level metrics and 20 variant-level metrics, plus genotype missing rate, ME rate, and genotype discordance rate, resulting in a comprehensive evaluation of the performance of different methods. ME rate is found to be nearly linearly correlated with genotype errors [42–44], so it is a useful quality metric for variants with pedigree information. Low genotype missing rate has been considered as an indicator of high-quality variant call set as a variant with high genotype missing rate indicates poor genotyping or sequencing quality [45]. Also, high-quality variants would have the same genotypes generated by different genotyping technologies, such as sequencing and microarray. Thus, variant sequencing quality may be measured with the genotype discordance rate between microarray and sequencing. One challenge with this approach is that genotypes generated by microarray are usually available for only a small proportion of variants in the whole genome, especially for common and known variants, so genotype discordance rate cannot be used to show the quality of the entire variant call-set. Another frequently used variant quality metric is the Ti/Tv ratio [46–49]. It is expected to be around 2.0 for WGS data [23]. That is because transitions occur more frequently according to molecular mechanisms, although the number of transversions is twice as many as transitions. Previous studies found that mitochondrial DNA and some non-human DNA sequences might be biased towards transitions or transversions [50,51]. In this study, we only computed the Ti/Tv ratio for each QC method using the same human variant call set excluding mitochondria, in order to achieve an unbiased evaluation of all methods.

The main advantage of our approach over the traditional filtering approach is that our method does not attempt to classify variants with ambiguous sequencing quality (undetermined variants) using filters. It is difficult to determine the quality of variants using filters if their QC metrics (e.g., genotype missing rate) are close to the thresholds. Hence, ForestQC avoids a limitation of the traditional filtering approaches that determine the quality of every variant using filters, which may exclude some of the high-quality variants from the downstream analysis. We did not compare our approach with the traditional filtering approach used in GWAS that removes variants according to HWE p-values, ME rates, and genotype missing rates. One main reason is that the performance of this approach changes dramatically depending on filters and their thresholds, and there are numerous different thresholds of filters, as well as many combinations of filters that could be tested. Another reason is that its performance could be arbitrarily determined depending on the filters we use. For example, if one filter is to remove any variants having more than zero Mendel errors, the ME rate of high-quality variants would be zero, but we may be removing many other high-quality variants. In this study, we checked the accuracy of a filtering approach based on ABHet as ABHet is often used in performing QC of NGS data and is an important indicator for variant quality [26,52,53]. Also, as this approach is not based on standard QC metrics such as genotype missing rate, its performance is independent of those metrics, unlike the standard filtering approaches. We showed that our approach outperformed the ABHet approach as the high-quality variants from ForestQC have better quality than those from ABHet, regardless of the similar total number of high-quality variants, in terms of ME rate, missing rate, genotype discordance rate, and Ti/Tv ratio in the BP and PSP dataset.

Although our approach is similar to VQSR as both approaches train machine learning classifiers to predict the quality of variants, they have a few differences. First, our approach trains the model using high-quality and low-quality variants detected from sequencing data on which quality control is performed, while VQSR uses variants in existing databases, such as HapMap and 1000 genomes, as its training set. As VQSR uses previously known variants for model training, high-quality variants from VQSR are likely to contain more known (and likely to be common) variants than novel (and rare) variants. We showed in both WGS datasets that VQSR did indeed identify more common and known SNVs and indels as high-quality variants than ForestQC. This may not be a desirable outcome for some sequencing studies if one of their main goals is to identify rare and novel variants not captured in chips. Another difference between ForestQC and VQSR is the set of features used in the classifiers. While both methods use features related to sequencing depth and genotyping quality, VQSR uses some features calculated explicitly by GATK software, while ForestQC uses quality information reported in the standard VCF file. This suggests that our method is more generalizable than VQSR as it can be applied to VCF files generated from variant callers other than GATK. The last difference is the machine learning algorithms that ForestQC and VQSR use. ForestQC trains a random forest classifier while VQSR trains a Gaussian Mixture model. And we found that ForestQC was much faster than VQSR. (S11 Table).

In addition to SNVs, we applied ForestQC to indels in both WGS datasets and found that indels had much lower sequencing quality than do SNVs as the fraction of high-quality indels detected by ForestQC was considerably smaller than that of SNVs. This is somewhat expected because indel or structural variant calling is much more complicated than SNV calling from sequencing data, and some of them are likely to be false-positives [54,55]. It is, however, important to note that VQSR classifies many more indels as high-quality variants than does ForestQC or ABHet, but those high-quality indels from VQSR may not have high sequencing quality. We showed that high-quality indels from VQSR had similar Mendelian error rate to that without performing QC, indicating the poor performance of VQSR on indels. VQSR considers indels from Mills gold standard call set [37] as true-positives. Although those indels might represent true variant sites, it does not necessarily mean that genotyping on those sites is accurate. Therefore, genetic studies need to perform stringent QC on indels to remove those erroneous calls and not to have false-positive findings in their downstream analysis.

We found that the performance of VQSR was improved dramatically in the BP dataset when we considered SNVs in Omni genotyping array to have both true and false-positive sites, compared with when they were assumed to have all true sites. We, however, did not observe this performance enhancement in the PSP dataset. This suggests that users may need to try different parameter settings to obtain optimal results from VQSR for specific sequencing datasets they analyze. Another issue with VQSR and also with ABHet is that some high-quality SNVs or indels have high genotype missing rate and ME rate, which may not be suitable for the downstream analysis such as association analysis. Thus, those variants need to be filtered out separately, which means users may need to perform an additional filtering step in addition to applying VQSR and ABHet to the dataset. As the filtering step is incorporated in ForestQC, our method does not have this issue.

Our approach is an extension of a previous approach that uses a logistic regression model to predict the quality of variants in the BP dataset [30]. While our approach is similar to the previous approach in that they both combine filtering and classification approaches, ForestQC uses a random forest classifier that has higher accuracy than a logistic regression model, according to our simulation results. It includes more low-quality variants for model training, leading to predictions with fewer biases. ForestQC also includes more features than the previous approach as well as more filters to improve the quality of variants. Additionally, compared with the previous approach, ForestQC is more user-friendly and generalizable because users can choose or define different features and filters and tune the parameters according to their research goals.

We want to note that in addition to applying ForestQC, one may do variant calling with high-quality reference genome and accurate variant callers to obtain accurate variant call-sets. As we know, it is crucial to choose a state-of-art variant caller to minimize errors and biases in variant calling. Also, the quality of the reference genome may have an impact on the quality of the resulting variant call-sets [56]. The higher the quality of the reference genome is, the fewer low-quality calls in the variant call-sets are expected. If the quality of the reference genome is expected to be low, we suggest users modify filters or features in ForestQC. For instance, users may want to introduce new features describing the quality of the reference genome, such as an indicator of whether a mutation site is in the high-confidence regions of the reference genome. Then, ForestQC may learn how this information affects variant quality during training, and the performance of the random forest classifier may be improved based on this information.

ForestQC is efficient, modularized, and flexible with the following features. First, users are allowed to change thresholds for filters as needed. This is important because filters that are stringent for one dataset may not be stringent for another dataset. For example, variants from sequence data with a small sample size (e.g., < 100) may not have large enough statistical power to have significant HWE p-values, so higher p-value thresholds should be used, compared with studies with larger sample size. If filters are not stringent enough, there may be many low-quality variants, and ForestQC would train a very stringent classifier, leading to the possible removal of high-quality variants. On the contrary, if the filters are too stringent, there would be too few high-quality variants or low-quality variants, which would lower the accuracy of our random forest classifier. In this study, after the filtering step, 4.39% of SNVs and 15.72% of indels in the BP dataset, and 5.06% of SNVs and 15.66% of indels in the PSP dataset, were determined as low-quality variants. Empirically, we suggest filters for ForestQC such that after the filtering step, a fraction of low-quality variants is about 4–16%. Usually, we recommend the default parameter settings, which are the same sets of filters and features described in this paper. The selection of threshold values for these filters is based on our previous study for WGS data of extended pedigrees for bipolar disorder [30]. Second, users are allowed to use self-defined filters and features provided that they specify values for those new filters and features at each variant site, and our software also allows users to remove existing filters and features. As there may be filters and features that capture the sequencing quality of variants more accurately than the current set of filters and features, this option allows users to improve ForestQC further. For example, users can employ mappability, strand bias, and micro-repeats as features, instead of sequencing depth and genotyping quality used in this study, because DP and GQ might penalize disease-causing variants with low coverage. Also, if users want to obtain more variants after QC, they may lower the standard for high-quality variants, that is, increase the threshold values of ME or missing rate for determining high-quality variants. Third, ForestQC generates the probability of each undetermined variant being a high-quality variant. This probability needs to be higher than a certain threshold for an undetermined variant to be predicted to be high-quality. It can also be used to analyze the sequencing quality of individual variants. If studies find that a particular undetermined variant is associated with a phenotype, they may consider checking whether its probability of being a high-quality variant is high enough. Lastly, ForestQC allows users to change the probability threshold for determining whether each undetermined variant is high-quality or low-quality. Users may lower this threshold if they are interested in obtaining more high-quality variants at the cost of including more low-quality variants.

## Materials and methods

### ForestQC

ForestQC consists of two approaches: a filtering approach and a machine learning approach based on a random forest algorithm.

#### Filtering

Given a variant call set from next-generation sequencing data, ForestQC first applies several stringent filters to identify high-quality, low-quality, and undetermined variants. High-quality variants are ones that pass all filters, while low-quality variants fail any of them (S1 and S2 Tables). The undetermined variants are variants that neither pass filters for high-quality variants nor fail filters for low-quality variants. We use the following filters in the filtering step.

- Mendelian error (ME) rate. The Mendelian error occurs when a child’s genotype is inconsistent with genotypes from parents. ME rate is calculated as the number of ME among all trios divided by the number of trios for a given variant. Note that this statistic is only available for family-based data.
- Genotype missing rate. This is the proportion of missing alleles in each variant.
- Hardy-Weinberg equilibrium (HWE) p-value. This is a p-value for hypothesis testing whether a variant is in Hardy-Weinberg equilibrium. Its null hypothesis is that the variant is in Hardy-Weinberg equilibrium. We use the algorithm from open-source software, VCFtools [57], for the calculation of Hardy-Weinberg equilibrium p-value.
- ABHet. This is the allele balance for heterozygous calls. ABHet is calculated as the number of reference reads from individuals with heterozygous genotypes divided by the total number of reads from such individuals, which is supposed to be 0.50 for high-quality bi-allelic variants. For variants in chromosome X, we only calculate ABHet for females.

#### Random forest classifier

Random forest is a machine learning algorithm that runs efficiently on large datasets with high accuracy [34]. Briefly, random forest builds several randomized decision trees, each of which is trained to classify the input objects. For the classification of a new object, the fitted random forest model passes the input vector down to each of the decision trees in the forest. Each decision tree has its classification result, and then the forest would output the classification that the majority of the decision trees make. To balance efficiency and accuracy, we train a random forest classifier using 50 decision trees (S2 Fig) and a probability threshold of 50% (S3 Fig).

To train random forest, we use high-quality and low-quality variants identified from the previous filtering step as a training dataset, after balancing their sample size by random sampling. Normally, high-quality variants are much more numerous than low-quality variants, so we randomly sample from high-quality variants with the sample size of low-quality variants. Hence, the sample size of the balanced training set would be twice as large as the sample size of low-quality variants. We also need features in training a random forest, which characterize datasets, and we use the following features.

- Mean and standard deviation of depth (DP) and genotyping quality (GQ). The depth and genotyping quality values are extracted from DP and GQ fields of each sample in VCF files, respectively, and mean and standard deviation are calculated over all samples for each variant.
- Outlier depth and outlier genotype quality. These are the proportions of samples whose DP or GQ is lower than a particular threshold. We choose this threshold as the first quartile value of all DP or GQ values of variants on chromosome 1. We use DP and GQ of variants on only chromosome 1 to reduce the computational costs.
- GC content: We first split a reference genome into windows with a size of 1,000 bp and calculate GC content for each window as (# of G or C alleles) / (# of A, G, C, or T alleles). Then, each variant is assigned a GC content value according to its position in the reference genome.

After training random forest with the training dataset using the above features, we next use the fitted model to make predictions on undetermined variants on being high-quality variants. Undetermined variants with the predicted probability of being high-quality larger than 50% are labeled as predicted high-quality variants. Then the predicted high-quality variants and high-quality variants from the previous filtering step are combined as the final set of high-quality variants. We apply the same procedure to identify low-quality variants.

### Comparison of different machine learning algorithms

We compare eight different machine learning algorithms to identify the best algorithm used for ForestQC. They are 1) k-nearest neighbors for supervised two-class classification (eight threads); 2) logistic regression (eight threads); 3) single support vector machine with Gaussian kernel function and penalty parameter C of 1.0 (one thread); 4) random forest with 50 trees (eight threads); 5) naïve Bayes without any prior probabilities of the classes (one thread); 6) artificial neural network with sigmoid function as activation function (eight threads). It has one hidden layer with ten units; 7) AdaBoost with 50 estimators and learning rate of 1.0, which uses SAMME.R real boosting algorithm (one thread); 8) and quadratic discriminant analysis without any prior on classes. Its regularization is 0, and its threshold for rank estimation is 1e-4 (one thread). Other parameters of these machine learning algorithms are the default, as described in the documentation of the Python scikit-learn package [58]. All learning algorithms use the seven features as mentioned earlier: mean and standard deviation of sequencing depth, mean and standard deviation of genotype quality, outlier depth, outlier quality, and GC content.

To test these eight machine learning algorithms, we obtain training and test datasets from the BP dataset, using filters described in S1 and S2 Tables. There are 21,248,103 high-quality SNVs and 2,257,506 high-quality indels while there are 1,100,325 low-quality SNVs and 624,965 low-quality indels. We sample 100,000 variants randomly from high-quality variants and 100,000 variants from low-quality variants to generate a training set. Similarly, 100,000 high-quality variants and 100,000 low-quality variants are randomly chosen from the rest of the variants to form a test set. Each machine learning model shares the same training and test sets. We train the machine learning models and measure training time at a training stage, and then test their accuracy and measure prediction time at a testing stage. We measure the runtime of each algorithm, which is the elapsed clock time between the start and end of each algorithm. To assess the performance of each algorithm, we compute the F1-score for the test set. F1-score is the harmonic average of precision and recall, which is calculated as $2∙precision∙\frac{recall}{\left(precision+recall\right)}$. The closer F1-score is to 1, the higher classification accuracy is. Recall is the fraction of true-positive results over all samples that should be given a positive prediction. Precision is the number of true-positive results divided by the number of positive results predicted by the classifier. We also measure the model accuracy using 10-fold cross-validation, as well as the area under the receiver operating characteristic curve.

### ABHet approach and VQSR

We compare ForestQC with two other approaches for performing QC on genetic variants. One is a filtering approach based on ABHet, and the other is a classification approach called VQSR from GATK software. For the ABHet approach, we consider variants with ABHet > 0.7 or < 0.3 as low-quality variants, and the rest as high-quality variants. We chose this threshold setting of ABHet (> 0.3 and < 0.7) because the ADSP project could not reliably confirm heterozygous calls with ABHet > 0.7 with Sanger sequencing [26]. We also exclude variants with small ABHet values (< 0.3) to ensure high quality. For GATK, we use recommended arguments as of 2018-04-04 [35]. For SNVs, VQSR takes SNVs in HapMap 3 release 3, 1000 Genome Project and Omni genotyping array as training resources, and dbSNP135 as known site resource. HapMap and Omni sites are considered as true sites, meaning that SNVs in these datasets are all true variants, while 1000 Genome Project sites are regarded as false sites, meaning that there could be both true and false-positive variants. The desired level of sensitivity of true sites is set to be 99.5%. In the BP dataset, we run VQSR version 3.5-0-g36282e4 with following annotations; quality by depth (QD), RMS mapping quality (MQ), mapping quality rank sum test (MQRankSum), read position rank sum test (ReadPosRankSum), fisher strand (FS), coverage (DP) and strand odds ratio (SOR) to evaluate the likelihood of true-positive calls. In the PSP dataset, we use VQSR version 3.2-2-gec30cee that uses all annotations above except for SOR and additional inbreeding coefficient (InbreedingCoeff) because variants in PSP dataset do not have the SOR annotation. For indels, VQSR takes indels in Mills gold standard call set [37] as a true training resource and dbSNP135 as a known site resource. The desired level of sensitivity of true sites is set to be 99.0%. We use VQSR version 3.5-0-g36282e4 with QD, DP, FS, SOR, ReadPosRankSum, and MQRankSum annotations to evaluate the likelihood of true-positive calls in the BP dataset, while we run VQSR version 3.2-2-gec30cee with the same annotations except for SOR and additional InbreedingCoeff for the PSP dataset.

### BP and PSP WGS datasets

The BP WGS dataset is for studying bipolar disorder whose average coverage is 36-fold. This study recruited individuals from 11 Colombia (CO) and 15 Costa Rica (CR) extended pedigrees in total. 454 subjects from 10 CO and 12 CR families are both whole-genome sequenced and genotyped with microarray. There are 144 individuals diagnosed with BP1 and 310 control samples that are unaffected or have non-BP traits. We use the highly scalable Churchill pipeline [59] to do the variant calling for the BP data set, where GATK-HaplotypeCaller 3.5-0-g36282e4 is used as the variant caller according to the GATK best practices [23], and the reference genome is HG19. After initial QC on individuals, five individuals are removed because of poor sequencing quality and possible sample mix-ups. Finally, 449 individuals are included in an analysis, resulting in 25,081,636 SNVs and 3,976,710 indels. 1,814,326 SNVs in the WGS dataset are also genotyped with microarray, which are used to calculate the genotype discordance rate. In this study, we use the BP dataset before any QC performed on genetic variants. In a previous study [30], genetic variants in the BP WGS dataset are first processed with VQSR and then filtered with a trained logistic regression model to remove variants with low quality.

The PSP WGS dataset is for studying progressive supranuclear palsy with an average coverage of 29-fold. 544 unrelated individuals are whole-genome sequenced, 518 of whom are also genotyped with microarray. Among them, 119 individuals have 547,644 SNPs, and 399 individuals have 1,682,489 SNPs genotyped with microarray, respectively. That 119 individuals would be excluded when calculating the genotype discordance rate in case of biases caused by fewer SNPs. There are 356 individuals diagnosed with PSP and 188 individuals as controls. Variant calling for the PSP dataset is performed using the Churchill pipeline, where GATK-HaplotypeCaller 3.2-2-gec30cee is used as the variant caller according to the GATK best practices, and the reference genome is HG19. Forty-nine samples are found to have high missing rate, high relatedness with other samples, or are diagnosed with diseases other than PSP, so they are removed. Next, we extract variant data with only 495 individuals with VCFtools. Monomorphic variants are then removed. After preprocessing, the PSP WGS dataset has 33,273,111 SNVs and 5,093,443 indels. There are 1,682,489 SNVs from 381 samples genotyped by both microarray and WGS, which are used for calculating genotype discordance rate.

### Performance metrics

Twenty-one sample-level metrics and twenty variant-level metrics are defined to measure the sequencing quality of the variant call-sets after quality control (S12 Table). Note that we do not show all sample-level metrics and variant-level metrics in the main text. Other metrics are available in supplemental materials. Variant-level metrics provide us with a summarized assessment report of the sequencing quality of a variant call set, such as total SNVs of the whole dataset. They are calculated based on the information of all variants in a variant call set. For example, the number and the proportion of multi-allelic SNVs are calculated for the entire dataset. On the other hand, sample-level metrics enable the inspection of the sequencing quality for sequenced individuals in a variant call set. For instance, we check the distribution of novel Ti/Tv or other quality metrics among all individuals in the study. Sample-level metrics are calculated for each sample, using its genotype information on all variants in the dataset. The distribution of those metrics across all individuals is shown as a box plot. For example, the number of SNV singletons on a sample level shows the distribution of the number of SNV singletons across all sequenced individuals. In this study, both sample-level and variant-level metrics are used to evaluate the sequencing quality of WGS variant datasets.

Additionally, we use genotype missing rate, ME rate and genotype discordance rate as variant quality metrics, which are computed using the entire variant call set. The definitions of genotype missing rate and ME rate have been described above. Note that ME rate is only available for family-based datasets, such as the BP dataset, so we do not calculate ME rate for the PSP dataset that only includes unrelated individuals. Genotype discordance rate is the proportion of individuals whose genotypes are inconsistent between next-generation sequencing and microarray. This metric can only be calculated with a subset of variants due to the limited number of variants genotyped by both sequencing and microarray. Note that microarray might also have biases in genotyping, leading to some limitations of genotype discordance rate. For example, microarray usually genotype selected variants, primarily common and known variants, so genotype discordance rate is only available for these selected variants, and it cannot provide quality evaluation for all variants, especially rare variants. Genotype missing rate, ME rate and genotype discordance rate provide us with an accurate evaluation of variant quality because true-positive variants with high quality are very likely to have low values of these three metrics.

## Supporting information

S1 Fig Receiver operating characteristic (ROC) curves and area under the curve of eight machine learning models in (a) SNV classification and (b) indel classification.
(TIFF)

S2 Fig Relationship between the number of trees in random forest model and the performance of ForestQC. Relationship between the number of trees and (a) CPU time and (b) F1-score.
(TIFF)

S3 Fig Relationship between the probability threshold for predicting a variant to be high-quality and the precision of ForestQC. If the probability of a variant predicted to be high-quality is larger than the probability threshold, this variant would be labeled as a high-quality variant. Classification precision changes along with the probability threshold in (a) SNV classification and (b) indel classification. The precision of ForestQC is measured in F1-score.
(TIFF)

S4 Fig Overall quality of high-quality and low-quality variants in the BP dataset identified by ForestQC using ME rate as a filter or not. (a) The ME rate of high-quality SNVs. (b) The ME rate of low-quality SNVs. (c) The missing rate of high-quality SNVs. (d) The missing rate of low-quality SNVs. (e) The genotype discordance rate of high-quality SNVs. (f) The genotype discordance rate of low-quality SNVs. (g) The ME rate of high-quality indels. (h) The ME rate of low-quality indels. (i) The missing rate of high-quality indels. (j) The missing rate of low-quality indels. Data are represented as the mean ± SEM.
(TIFF)

S5 Fig Sample-level quality metrics of high-quality variants in the BP dataset identified by ForestQC using ME rate as a filter or not. (a) Total number of SNVs. (b) The number of SNVs found in dbSNP. (c) The number of SNVs not found in dbSNP. (d) Ti/Tv ratio of SNVs found in dbSNP. (e) Ti/Tv ratio of SNVs not found in dbSNP. (f) The total number of indels. (g) The number of indels found in dbSNP. (h) The number of indels not found in dbSNP. The version of dbSNP is 150.
(TIFF)

S6 Fig Overall quality of low-quality variants in the BP dataset detected by four different methods, including no QC applied, ABHet approach, VQSR, and ForestQC. (a) The ME rate, (b) the missing rate, and (c) the genotype discordance rate of low-quality SNVs. (d) The ME rate and (e) the missing rate of low-quality indels. Data are represented as the mean ± SEM.
(TIFF)

S7 Fig Sample-level quality metrics of high-quality variants in the BP dataset identified by four different methods, including no QC applied, ABHet approach, VQSR, and ForestQC. (a) Ti/Tv ratio of SNVs found in dbSNP. (b) The number of SNVs found in dbSNP. (c) The number of indels found in dbSNP. (d) The number of indels not found in dbSNP. The version of dbSNP is 150.
(TIFF)

S8 Fig Overall quality of rare variants (MAF < 0.03) and common variants (MAF ≥ 0.03) in the BP dataset. (a) The ME rate, (b) the missing rate, and (c) the genotype discordance rate of rare and common SNVs. (d) The ME rate and (e) the missing rate of rare and common indels. Data are represented as the mean ± SEM.
(TIFF)

S9 Fig Overall quality of high-quality variants identified from undetermined variants in the BP dataset processed by four different methods, including no QC applied, ABHet approach, VQSR, and ForestQC. (a) The ME rate, (b) the genotype discordance rate, and (c) the missing rate of high-quality SNVs predicted from undetermined SNVs. (d) The ME rate and (e) the missing rate of high-quality indels predicted from undetermined indels. Data are represented as the mean ± SEM.
(TIFF)

S10 Fig Sample-level quality metrics of high-quality variants identified from undetermined variants in the BP dataset processed by four different methods, including no QC applied, ABHet approach, VQSR, and ForestQC. (a) The total number of SNVs. (b) The number of SNVs found in dbSNP. (c) The number of SNVs not found in dbSNP. (d) Ti/Tv ratio of SNVs found in dbSNP. (e) Ti/Tv ratio of SNVs not found in dbSNP. (f) The total number of indels. (g) The number of indels found in dbSNP. (h) The number of indels not found in dbSNP. The version of dbSNP is 150.
(TIFF)

S11 Fig Selected sample-level quality metrics of high-quality variants in BP dataset identified by VQSR using “SOR” or not. (a) Ti/Tv ratio of SNVs not found in dbSNP, (b) the number of total SNVs, and (c) the number of total indels in the BP dataset processed with VQSR using “SOR” or not. SOR stands for StrandOddsRatio, which is a metric for strand bias measured by the Symmetric Odds Ratio test. The version of dbSNP is 150.
(TIFF)

S12 Fig Overall quality of low-quality variants in the PSP dataset detected by four different methods, including no QC applied, ABHet approach, VQSR, and ForestQC. (a) The missing rate and (b) the genotype discordance rate of low-quality SNVs. (c) The missing rate of low-quality indels. Data are represented as the mean ± SEM.
(TIFF)

S13 Fig Sample-level quality metrics of high-quality variants in PSP dataset identified by four different methods, including no QC applied, ABHet approach, VQSR, and ForestQC. (a) Ti/Tv ratio of SNVs found in dbSNP. (b) The number of SNVs found in dbSNP. (c) The number of indels found in dbSNP. (d) The number of indels not found in dbSNP. The version of dbSNP is 150.
(TIFF)

S14 Fig Overall quality of rare variants (MAF < 0.03) and common variants (MAF ≥ 0.03) in the PSP dataset. (a) The missing rate and (b) the genotype discordance rate of rare and common SNVs. (c) The missing rate of rare and common indels. Data are represented as the mean ± SEM.
(TIFF)

S15 Fig Overall quality of high-quality variants identified from undetermined variants in the PSP dataset processed by four different methods, including no QC applied, ABHet approach, VQSR, and ForestQC. (a) The missing rate and (b) the genotype discordance rate of high-quality SNVs predicted from undetermined SNVs. (c) The missing rate of high-quality indels predicted from undetermined indels. Data are represented as the mean ± SEM.
(TIFF)

S16 Fig Sample-level quality metrics of high-quality variants identified from undetermined variants in the PSP dataset processed by four different methods, including no QC applied, ABHet approach, VQSR, and ForestQC. (a) The total number of SNVs. (b) The number of SNVs found in dbSNP. (c) The number of SNVs not found in dbSNP. (d) Ti/Tv ratio of SNVs found in dbSNP. (e) Ti/Tv ratio of SNVs not found in dbSNP. (f) The total number of indels. (g) The number of indels found in dbSNP. (h) The number of indels not found in dbSNP. The version of dbSNP is 150.
(TIFF)

S17 Fig Feature importance of each feature in the random forest model of ForestQC applied to the BP and PSP datasets. **DP stands for sequencing depth.** GQ stands for genotyping quality. SD means standard deviation. Outlier DP or GQ means the proportion of samples having genotyping quality or sequencing depth lower than the first quartile of depth or genotyping quality in chromosome 1. GC stands for the GC content of a 1000-bp window where the variant is located. (a) Feature importance in SNV classification. (b) Feature importance in indel classification.
(TIFF)

S18 Fig : Pearson’s correlation coefficients between each pair of features in (a) the BP SNV dataset, (b) the PSP SNV dataset, (c) the BP indel dataset, and (d) the PSP indel dataset.
(TIFF)

S19 Fig Quality of high-quality SNVs identified by VQSR with two different settings of training resources and ForestQC. (a) Ti/Tv ratio of SNVs not found in dbSNP v150 and (b) the total number of SNVs in the BP and PSP dataset. (c)-(e) Average Mendelian error rate, average genotype missing rate, and average genotype discordance rate of high-quality SNVs in the BP dataset. Data are represented as the mean ± SEM. “Omni_Modified VQSR”: SNVs in the Omni chip array call set are considered to contain both true- and false-positive sites. “original VQSR”: SNVs in Omni chip array call set are considered to contain only true sites.
(TIFF)

S1 Table Thresholds of four filters for the selection of high-quality variants from the original dataset. (DOCX)

S2 Table Thresholds of four filters for the selection of low-quality variants from the original dataset. (DOCX)

S3 Table Accuracy of eight different machine learning algorithms. (DOCX)

S4 Table Variant-level quality metrics of variants in the BP dataset processed by ForestQC with different settings. (DOCX)

S5 Table Variant-level quality metrics of high-quality variants in the BP dataset processed by different methods. (DOCX)

S6 Table Rare variants and common variants in the BP dataset processed by different methods. (DOCX)

S7 Table Variant-level quality metrics of high-quality variants identified from undetermined variants in the BP dataset. (DOCX)

S8 Table Variant-level quality metrics of high-quality variants in the PSP dataset processed by four different methods. (DOCX)

S9 Table Rare variants and common variants in the PSP dataset processed by different methods. (DOCX)

S10 Table Variant-level quality metrics of high-quality variants identified from undetermined variants in the PSP dataset. (DOCX)

S11 Table Running time of ForestQC and VQSR in two datasets, measured in real time. (DOCX)

S12 Table Definitions of 23 metrics for sequencing quality control calculated for sample-level and variant-level. (DOCX)
