# Supplementary figures and images for: ForestQC: Quality control on genetic variants from next-generation sequencing data using random forest
Source: PLoS Comput Biol. 2019 Dec 18;15(12):e1007556. doi: 10.1371/journal.pcbi.1007556 (PMC6938691; doi:10.1371/journal.pcbi.1007556)

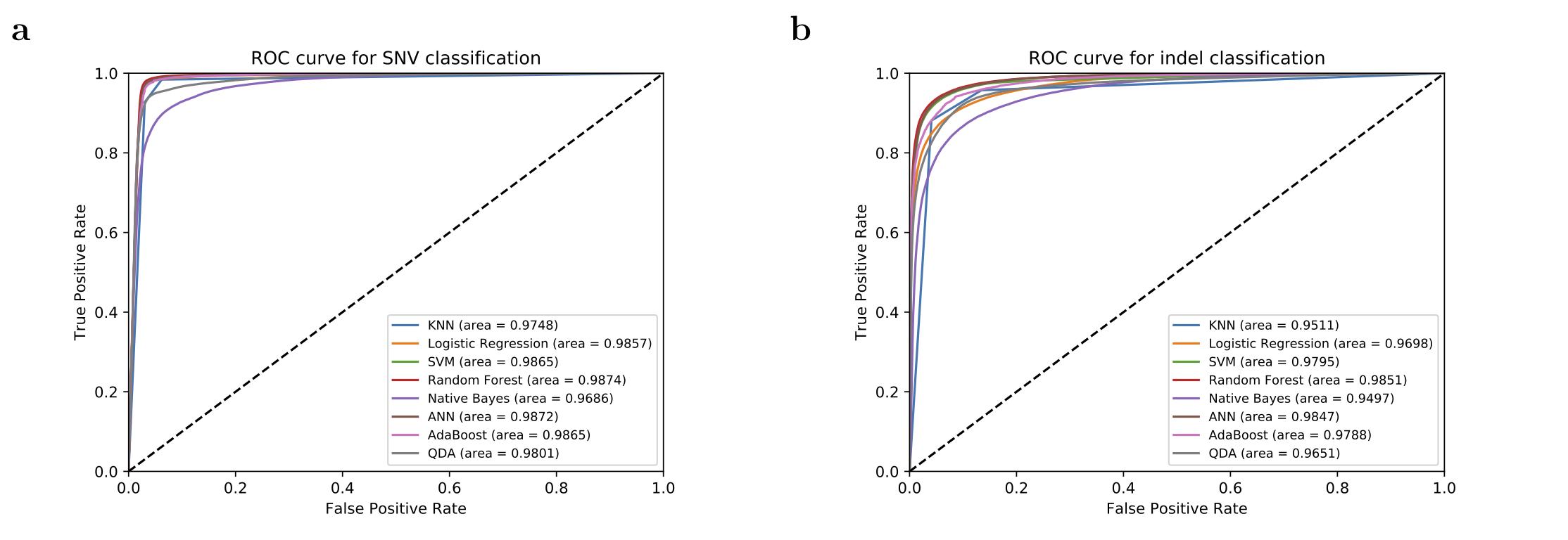

Supplement: S1 Fig — Receiver operating characteristic (ROC) curves and area under the curve of eight machine learning models in (a) SNV classification and (b) indel classification. (TIFF) [file pcbi.1007556.s001.tiff]

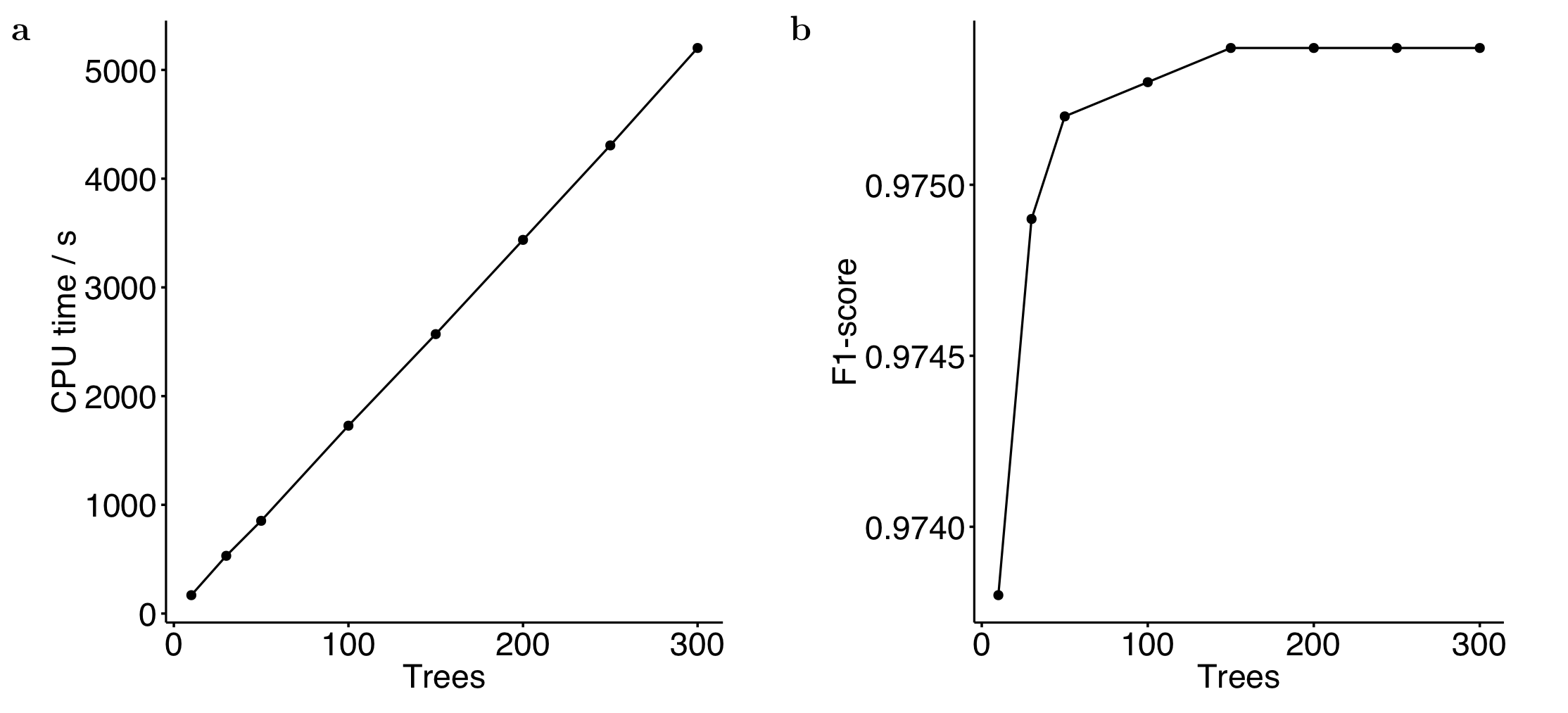

Supplement: S2 Fig — Relationship between the number of trees and (a) CPU time and (b) F1-score. (TIFF) [file pcbi.1007556.s002.tiff]

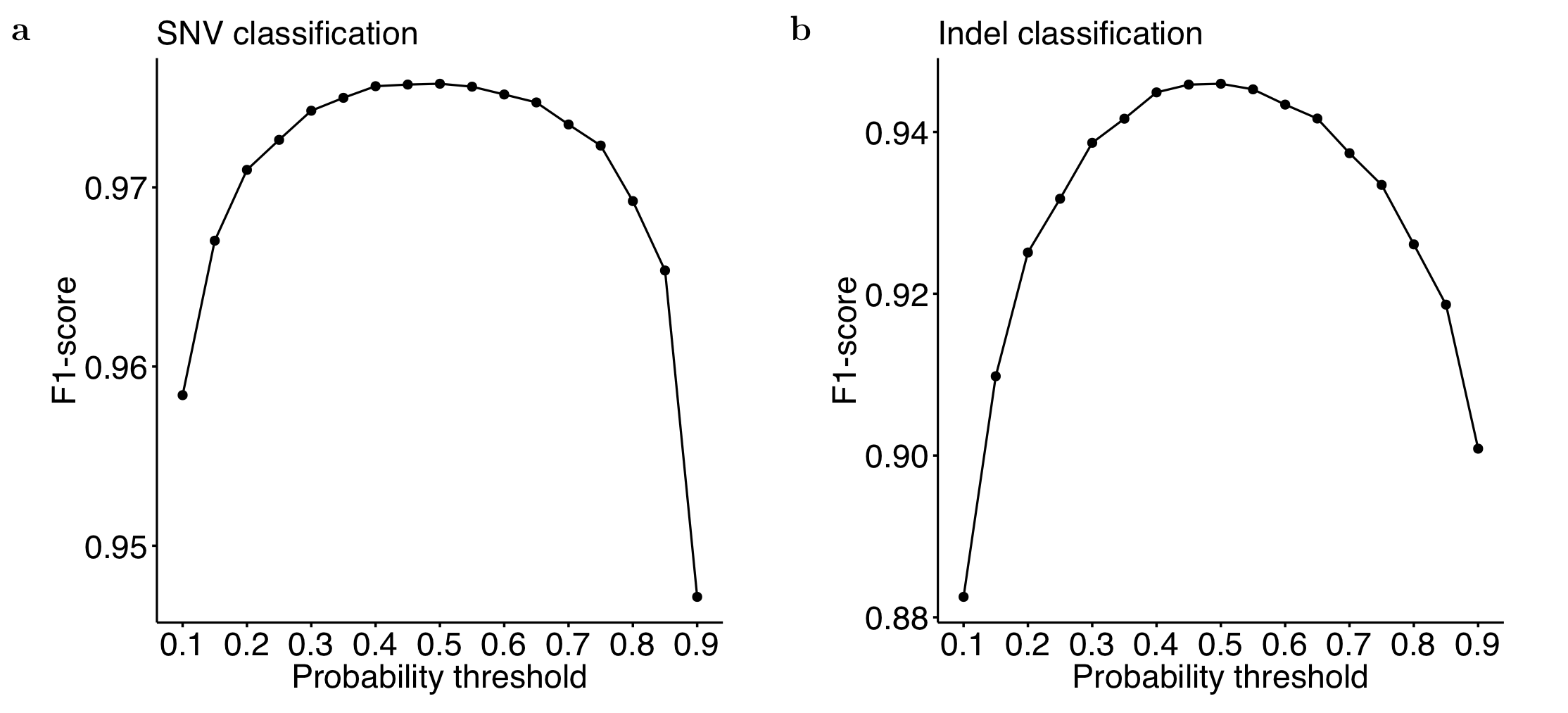

Supplement: S3 Fig — If the probability of a variant predicted to be high-quality is larger than the probability threshold, this variant would be labeled as a high-quality variant. Classification precision changes along with the probability threshold in (a) SNV classification and (b) indel classification. The precision of ForestQC is measured in F1-score. (TIFF) [file pcbi.1007556.s003.tiff]

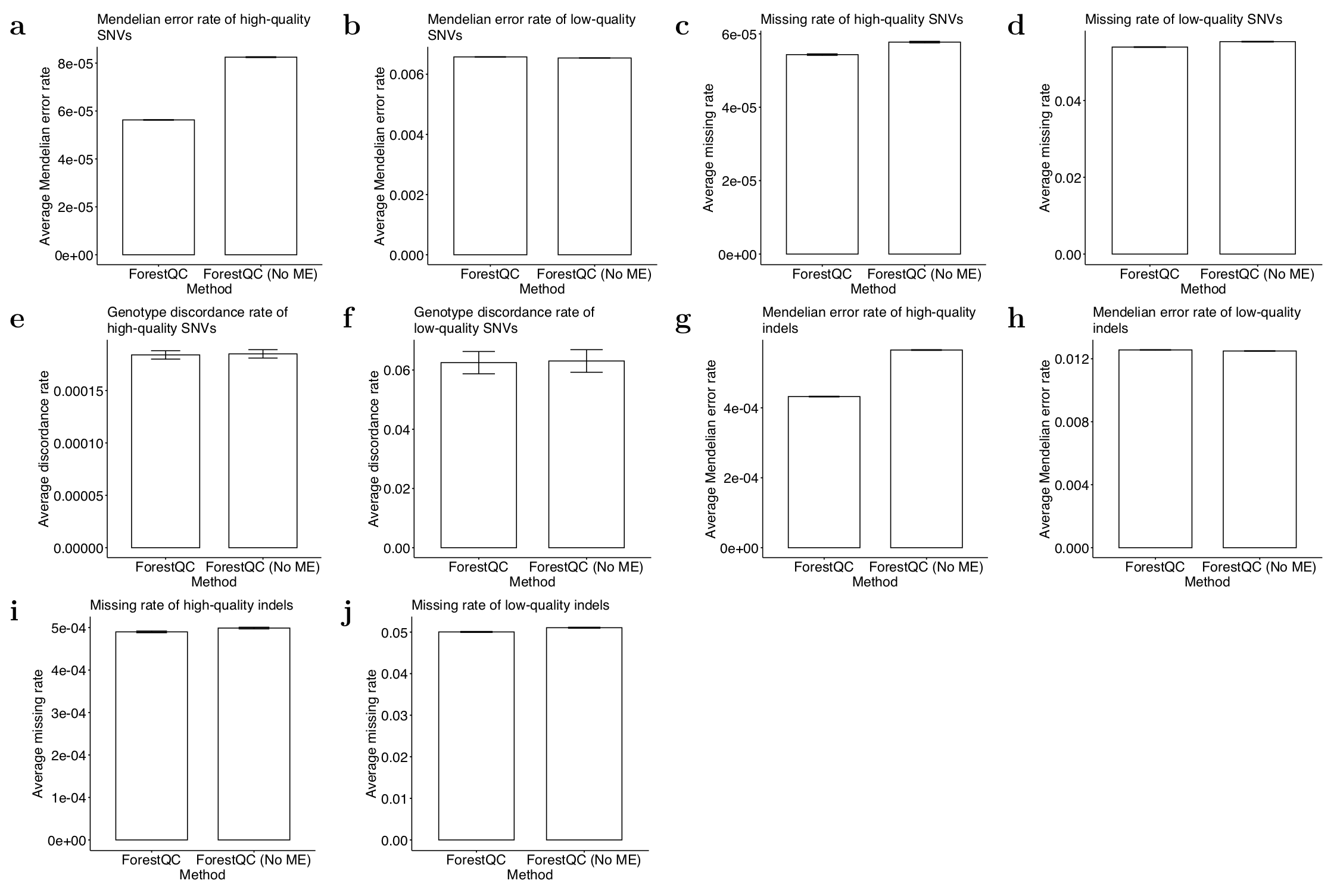

Supplement: S4 Fig — (a) The ME rate of high-quality SNVs. (b) The ME rate of low-quality SNVs. (c) The missing rate of high-quality SNVs. (d) The missing rate of low-quality SNVs. (e) The genotype discordance rate of high-quality SNVs. (f) The genotype discordance rate of low-quality SNVs. (g) The ME rate of high-quality indels. (h) The ME rate of low-quality indels. (i) The missing rate of high-quality indels. (j) The missing rate of low-quality indels. Data are represented as the mean ± SEM. (TIFF) [file pcbi.1007556.s004.tiff]

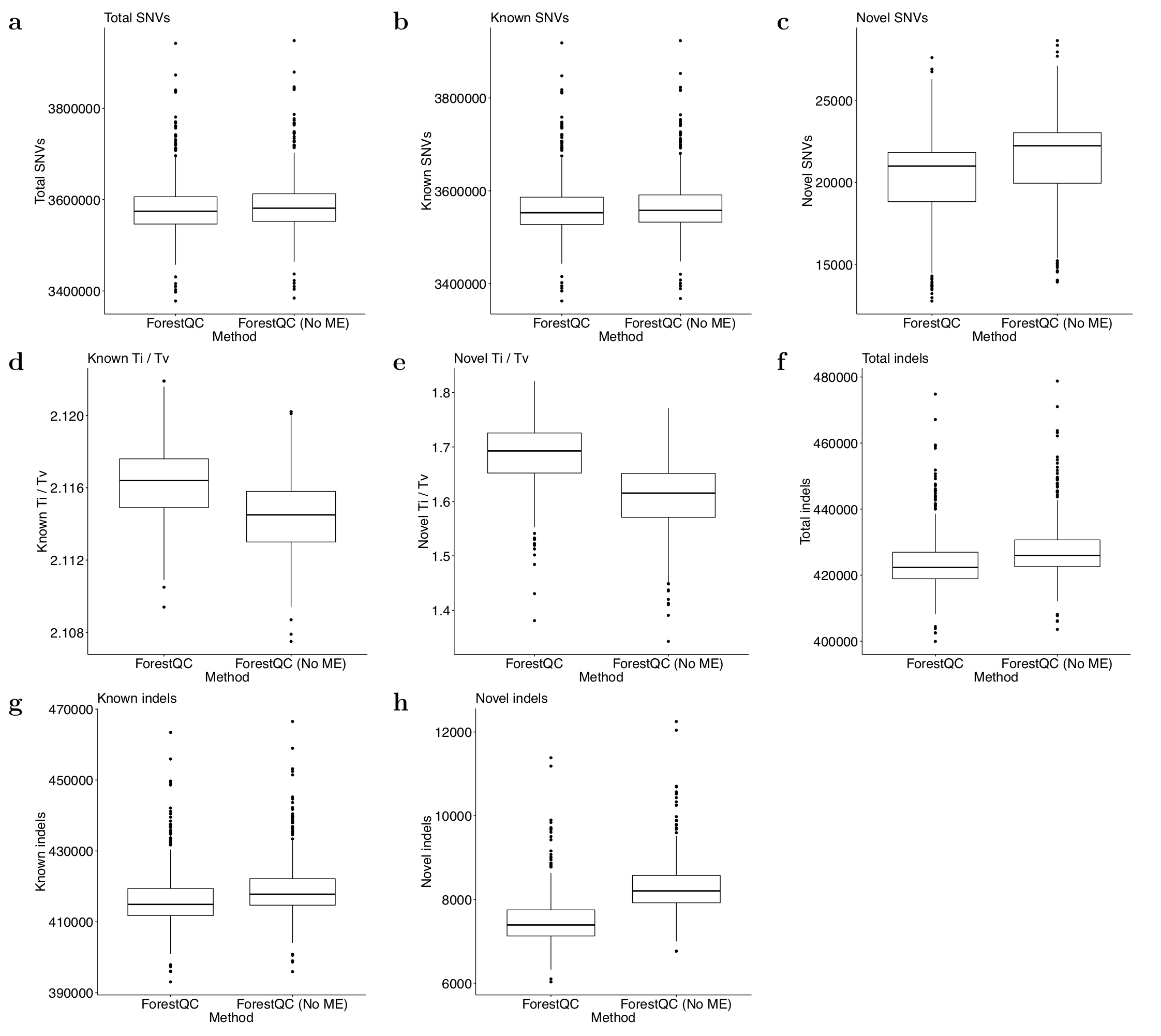

Supplement: S5 Fig — (a) Total number of SNVs. (b) The number of SNVs found in dbSNP. (c) The number of SNVs not found in dbSNP. (d) Ti/Tv ratio of SNVs found in dbSNP. (e) Ti/Tv ratio of SNVs not found in dbSNP. (f) The total number of indels. (g) The number of indels found in dbSNP. (h) The number of indels not found in dbSNP. The version of dbSNP is 150. (TIFF) [file pcbi.1007556.s005.tiff]

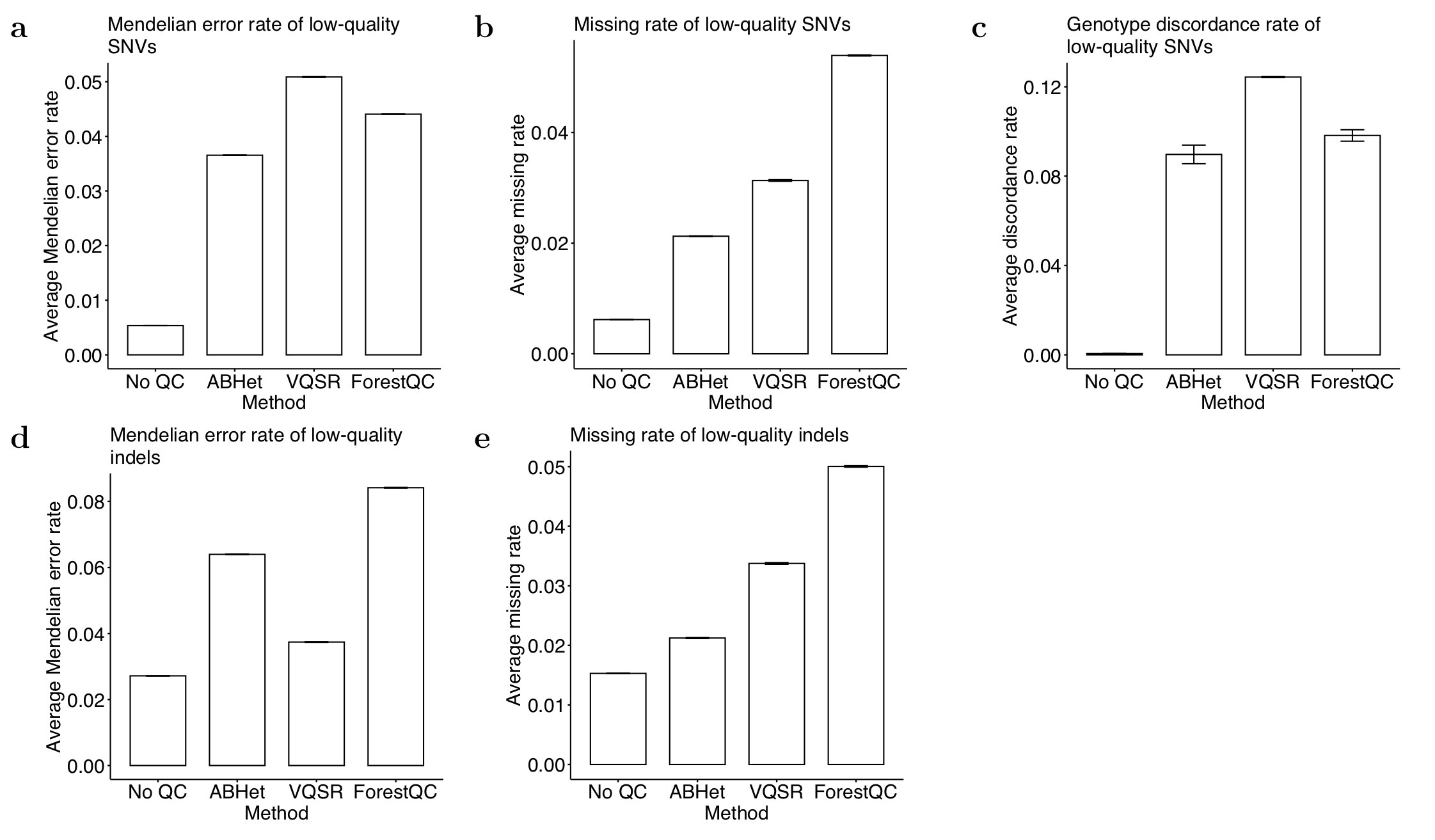

Supplement: S6 Fig — (a) The ME rate, (b) the missing rate, and (c) the genotype discordance rate of low-quality SNVs. (d) The ME rate and (e) the missing rate of low-quality indels. Data are represented as the mean ± SEM. (TIFF) [file pcbi.1007556.s006.tiff]

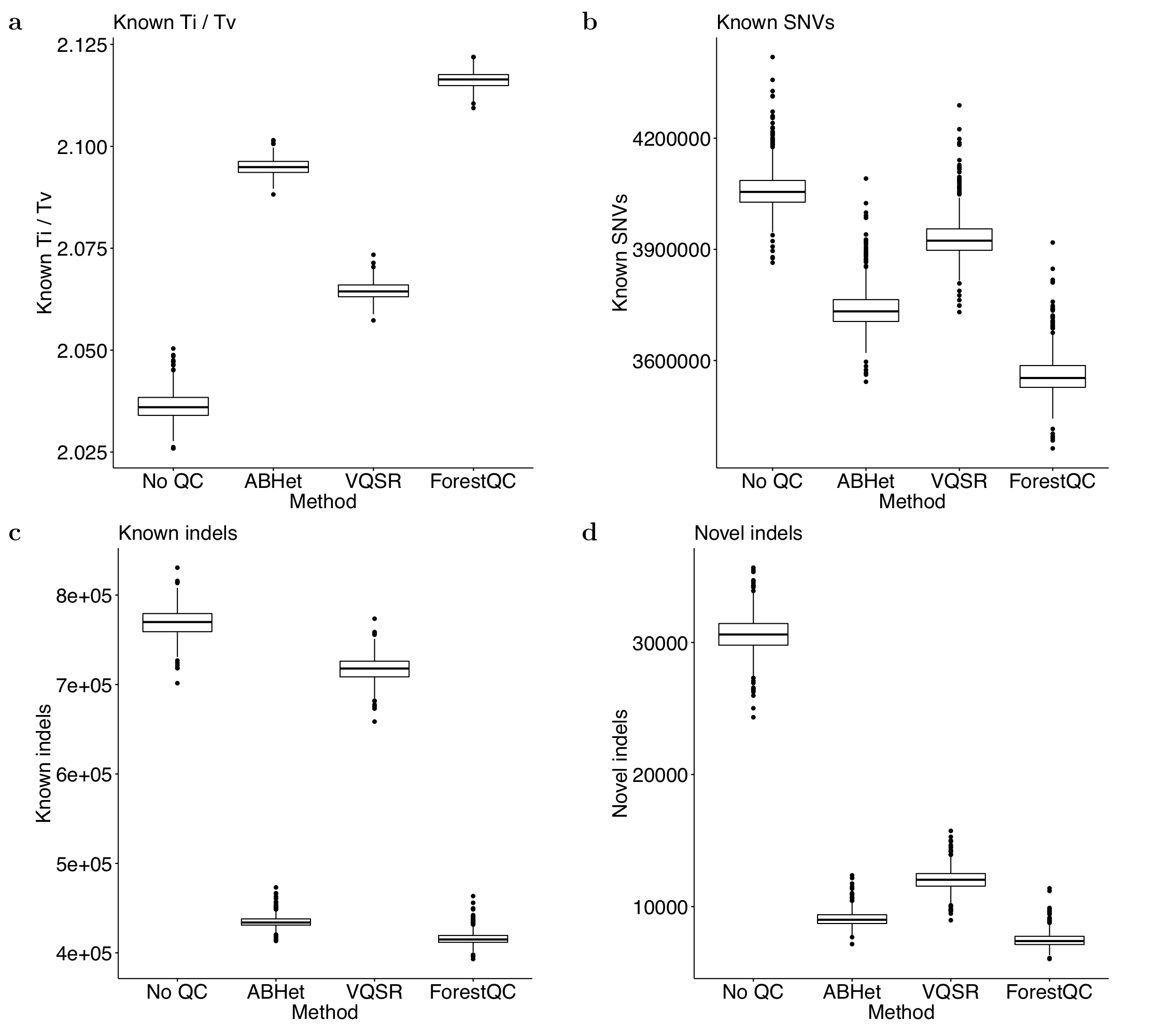

Supplement: S7 Fig — (a) Ti/Tv ratio of SNVs found in dbSNP. (b) The number of SNVs found in dbSNP. (c) The number of indels found in dbSNP. (d) The number of indels not found in dbSNP. The version of dbSNP is 150. (TIFF) [file pcbi.1007556.s007.tiff]

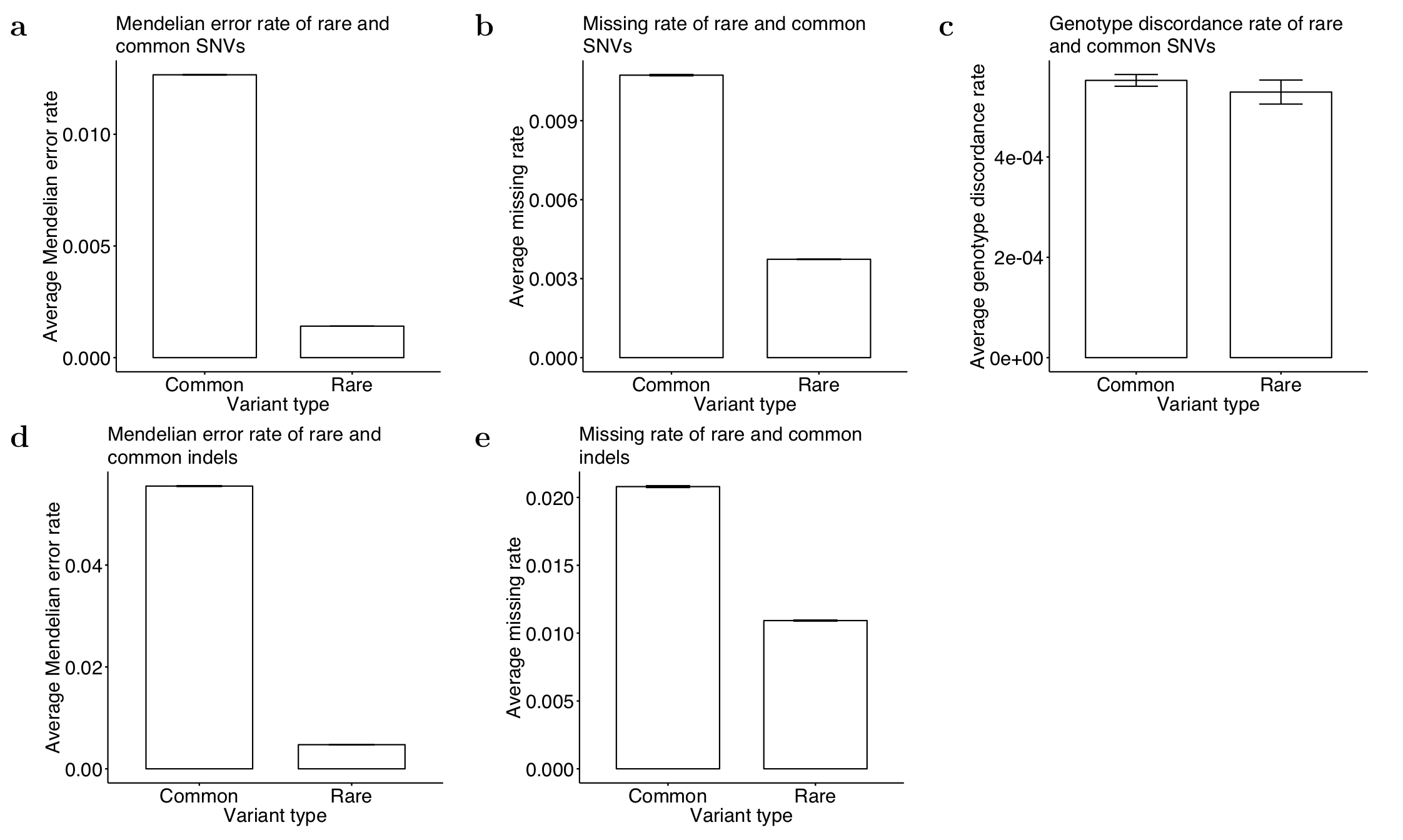

Supplement: S8 Fig — (a) The ME rate, (b) the missing rate, and (c) the genotype discordance rate of rare and common SNVs. (d) The ME rate and (e) the missing rate of rare and common indels. Data are represented as the mean ± SEM. (TIFF) [file pcbi.1007556.s008.tiff]

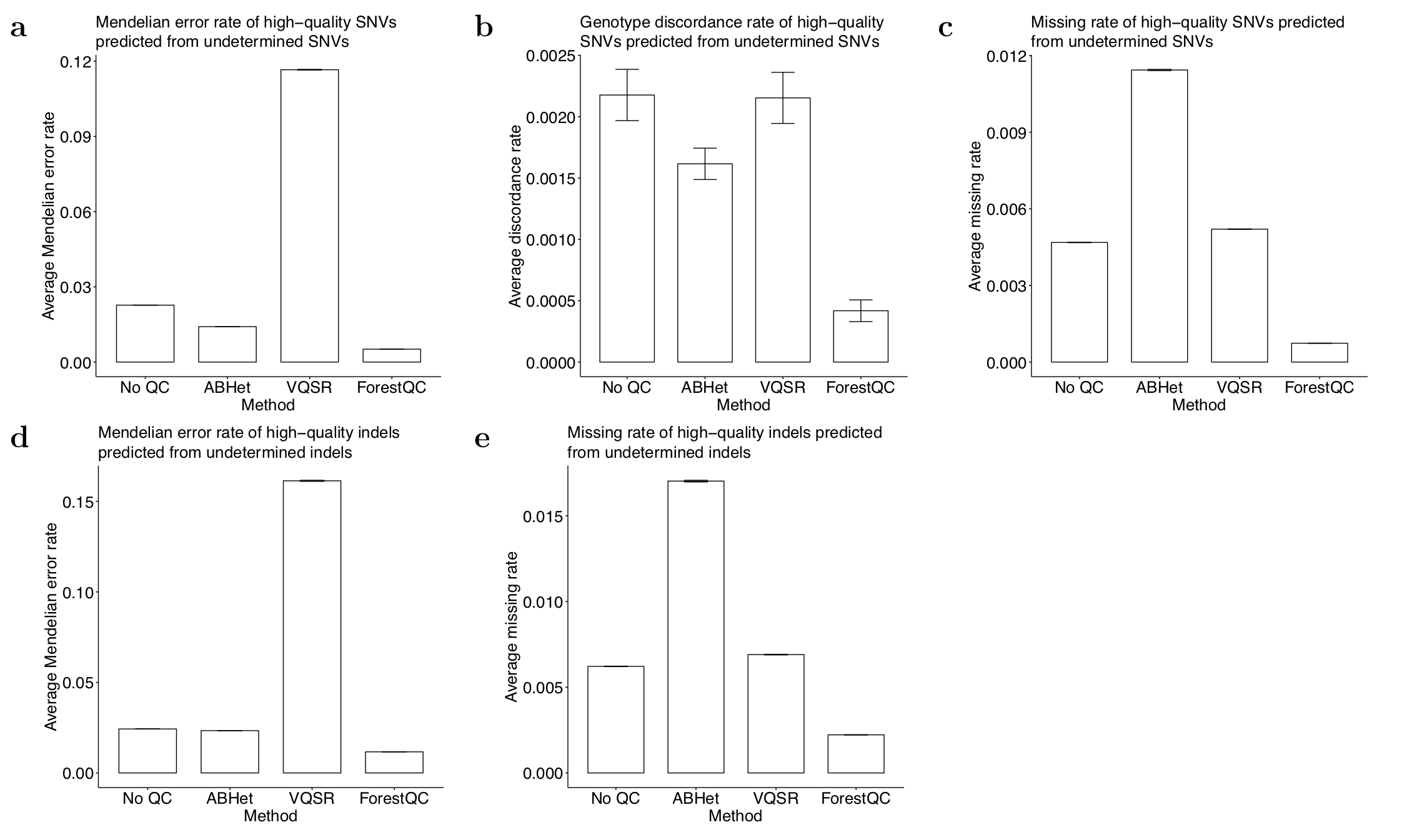

Supplement: S9 Fig — (a) The ME rate, (b) the genotype discordance rate, and (c) the missing rate of high-quality SNVs predicted from undetermined SNVs. (d) The ME rate and (e) the missing rate of high-quality indels predicted from undetermined indels. Data are represented as the mean ± SEM. (TIFF) [file pcbi.1007556.s009.tiff]

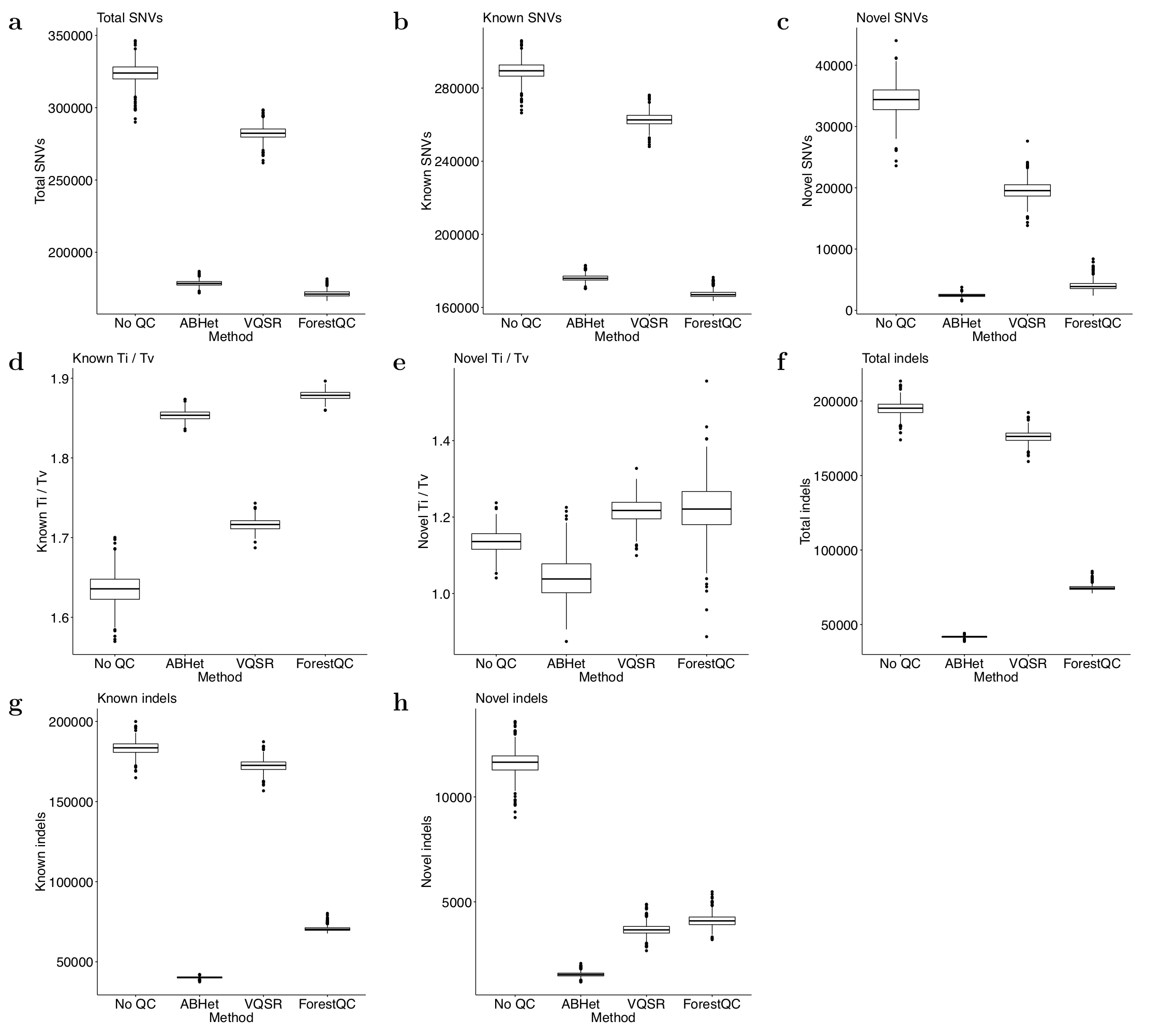

Supplement: S10 Fig — (a) The total number of SNVs. (b) The number of SNVs found in dbSNP. (c) The number of SNVs not found in dbSNP. (d) Ti/Tv ratio of SNVs found in dbSNP. (e) Ti/Tv ratio of SNVs not found in dbSNP. (f) The total number of indels. (g) The number of indels found in dbSNP. (h) The number of indels not found in dbSNP. The version of dbSNP is 150. (TIFF) [file pcbi.1007556.s010.tiff]

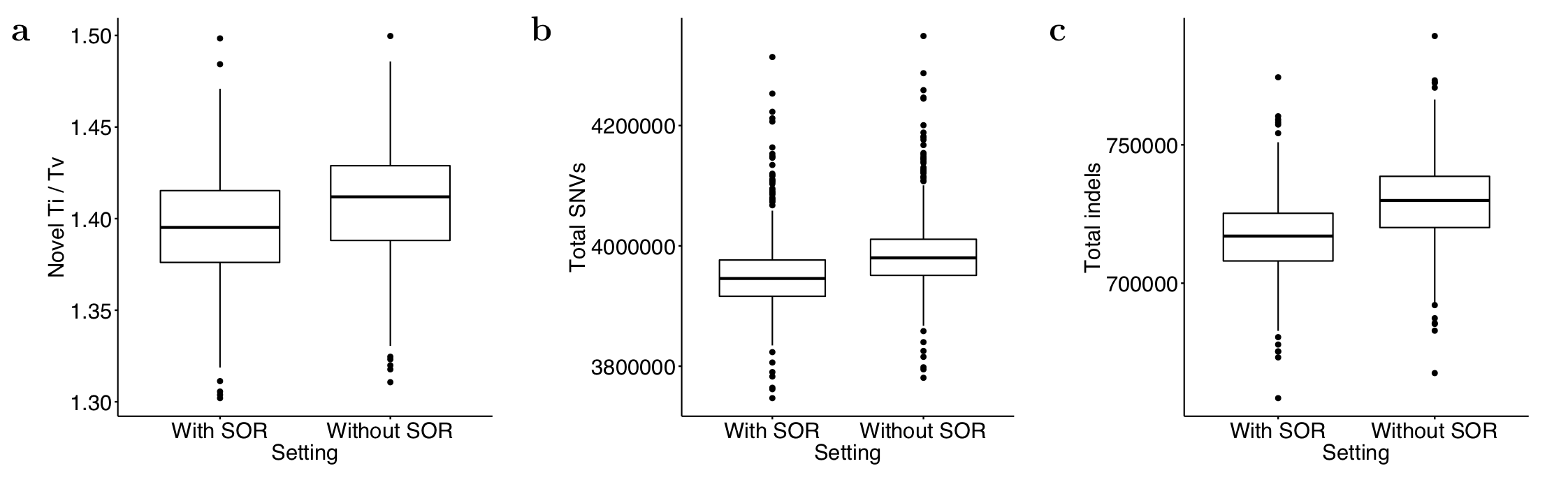

Supplement: S11 Fig — (a) Ti/Tv ratio of SNVs not found in dbSNP, (b) the number of total SNVs, and (c) the number of total indels in the BP dataset processed with VQSR using “SOR” or not. SOR stands for StrandOddsRatio, which is a metric for strand bias measured by the Symmetric Odds Ratio test. The version of dbSNP is 150. (TIFF) [file pcbi.1007556.s011.tiff]

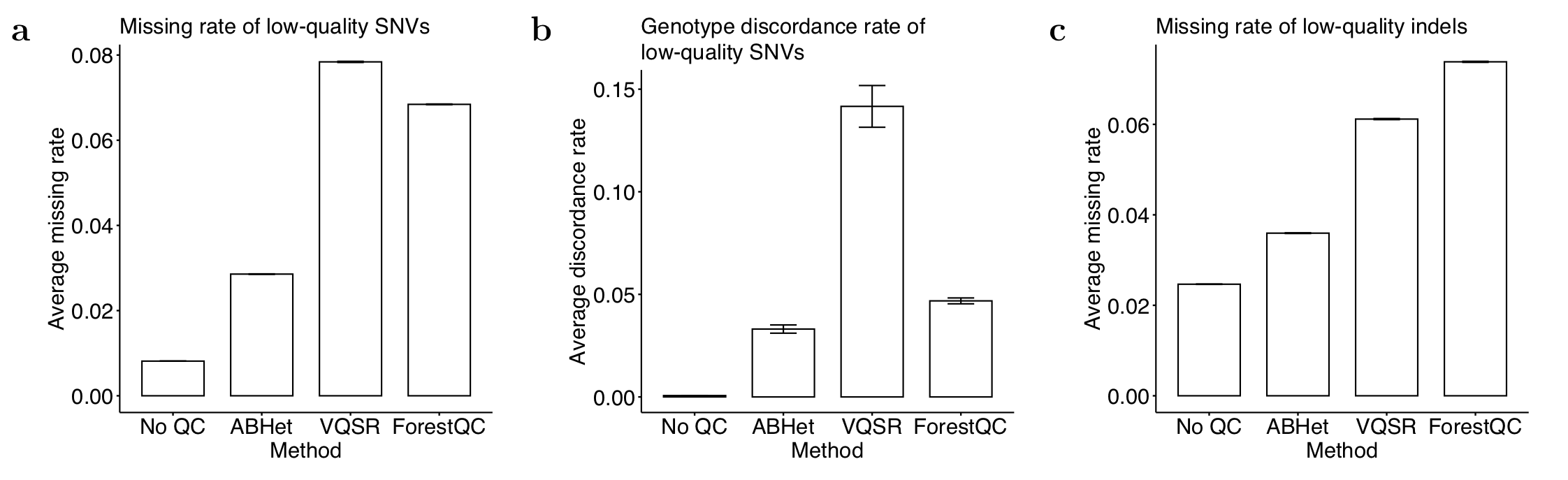

Supplement: S12 Fig — (a) The missing rate and (b) the genotype discordance rate of low-quality SNVs. (c) The missing rate of low-quality indels. Data are represented as the mean ± SEM. (TIFF) [file pcbi.1007556.s012.tiff]

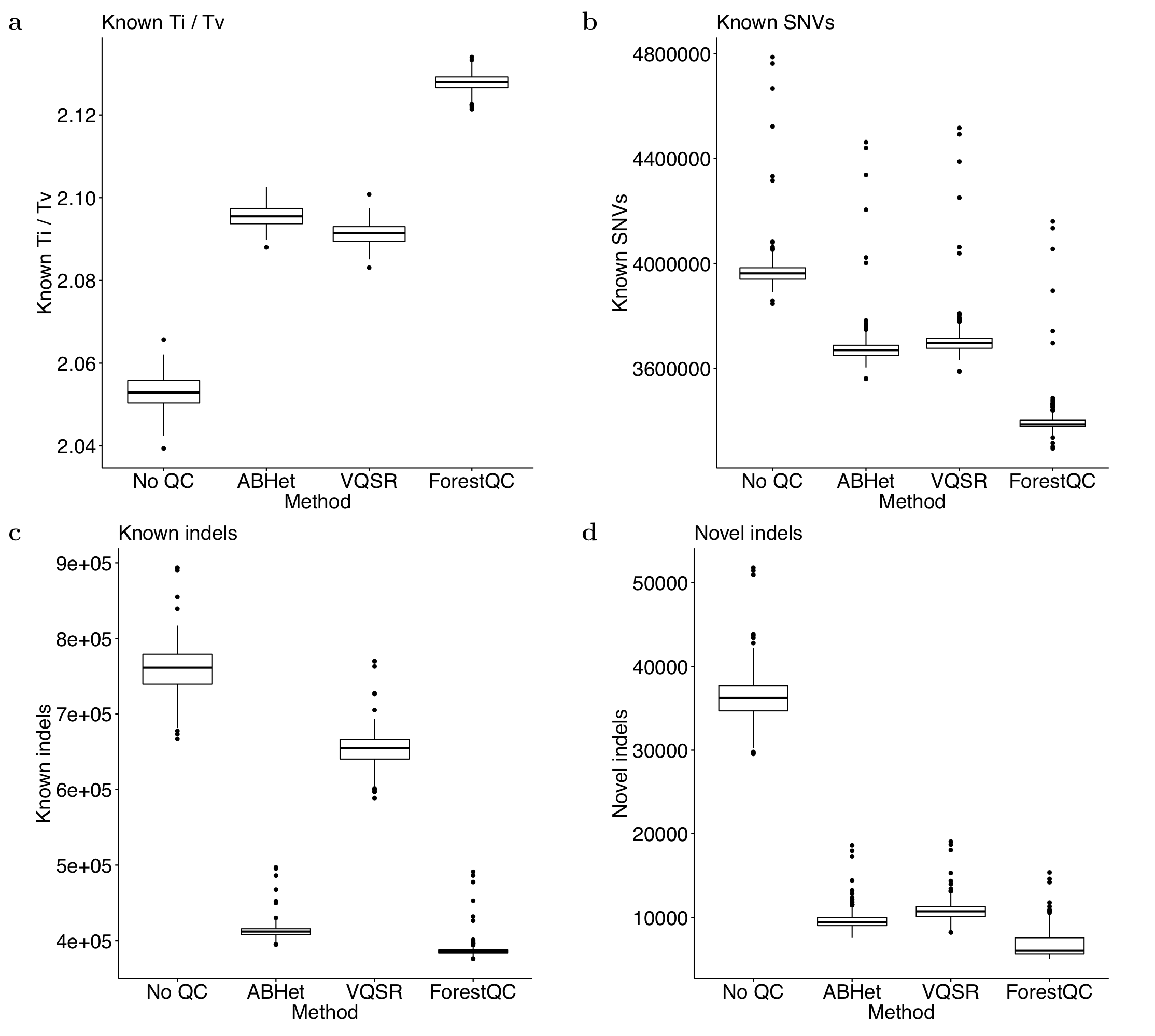

Supplement: S13 Fig — (a) Ti/Tv ratio of SNVs found in dbSNP. (b) The number of SNVs found in dbSNP. (c) The number of indels found in dbSNP. (d) The number of indels not found in dbSNP. The version of dbSNP is 150. (TIFF) [file pcbi.1007556.s013.tiff]

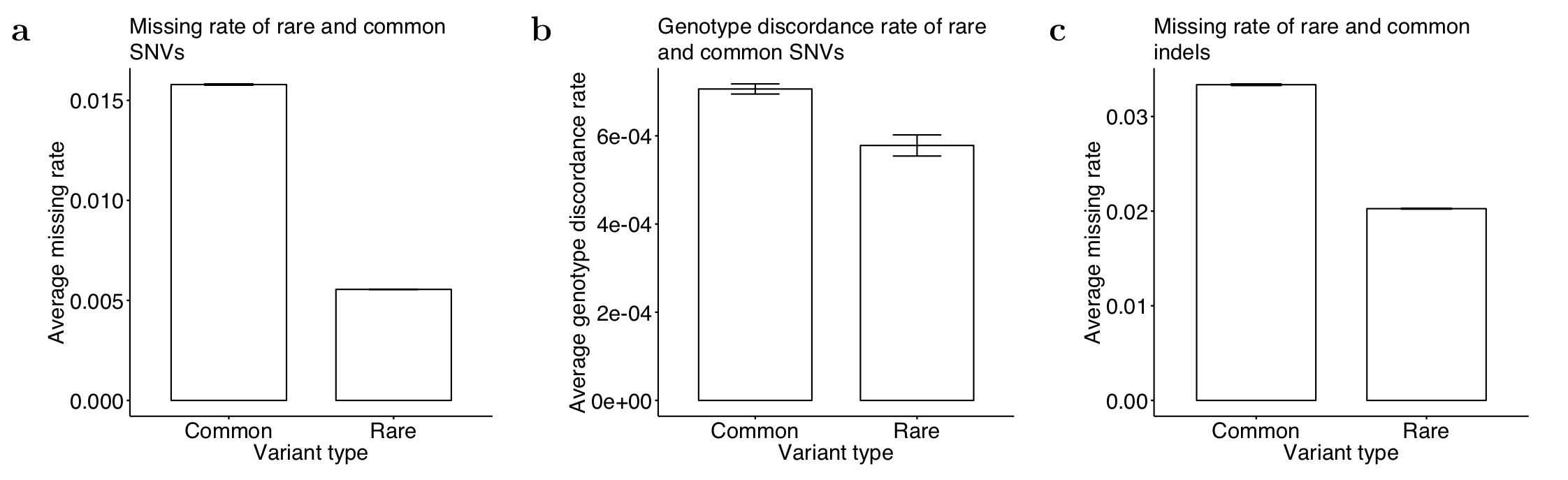

Supplement: S14 Fig — (a) The missing rate and (b) the genotype discordance rate of rare and common SNVs. (c) The missing rate of rare and common indels. Data are represented as the mean ± SEM. (TIFF) [file pcbi.1007556.s014.tiff]

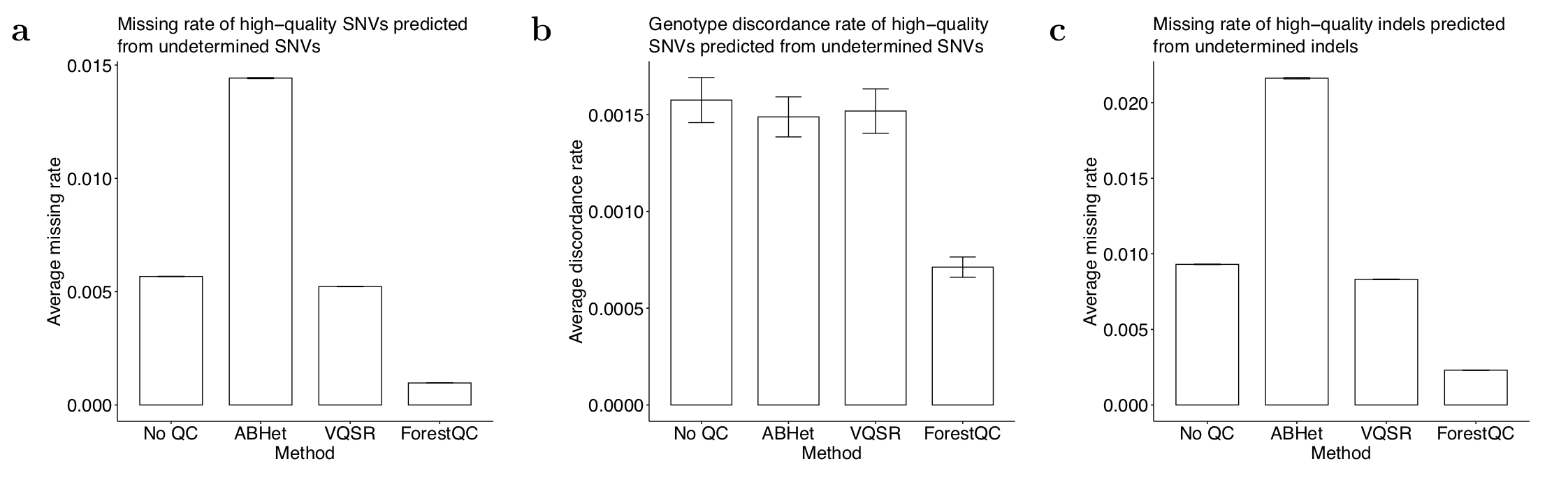

Supplement: S15 Fig — (a) The missing rate and (b) the genotype discordance rate of high-quality SNVs predicted from undetermined SNVs. (c) The missing rate of high-quality indels predicted from undetermined indels. Data are represented as the mean ± SEM. (TIFF) [file pcbi.1007556.s015.tiff]

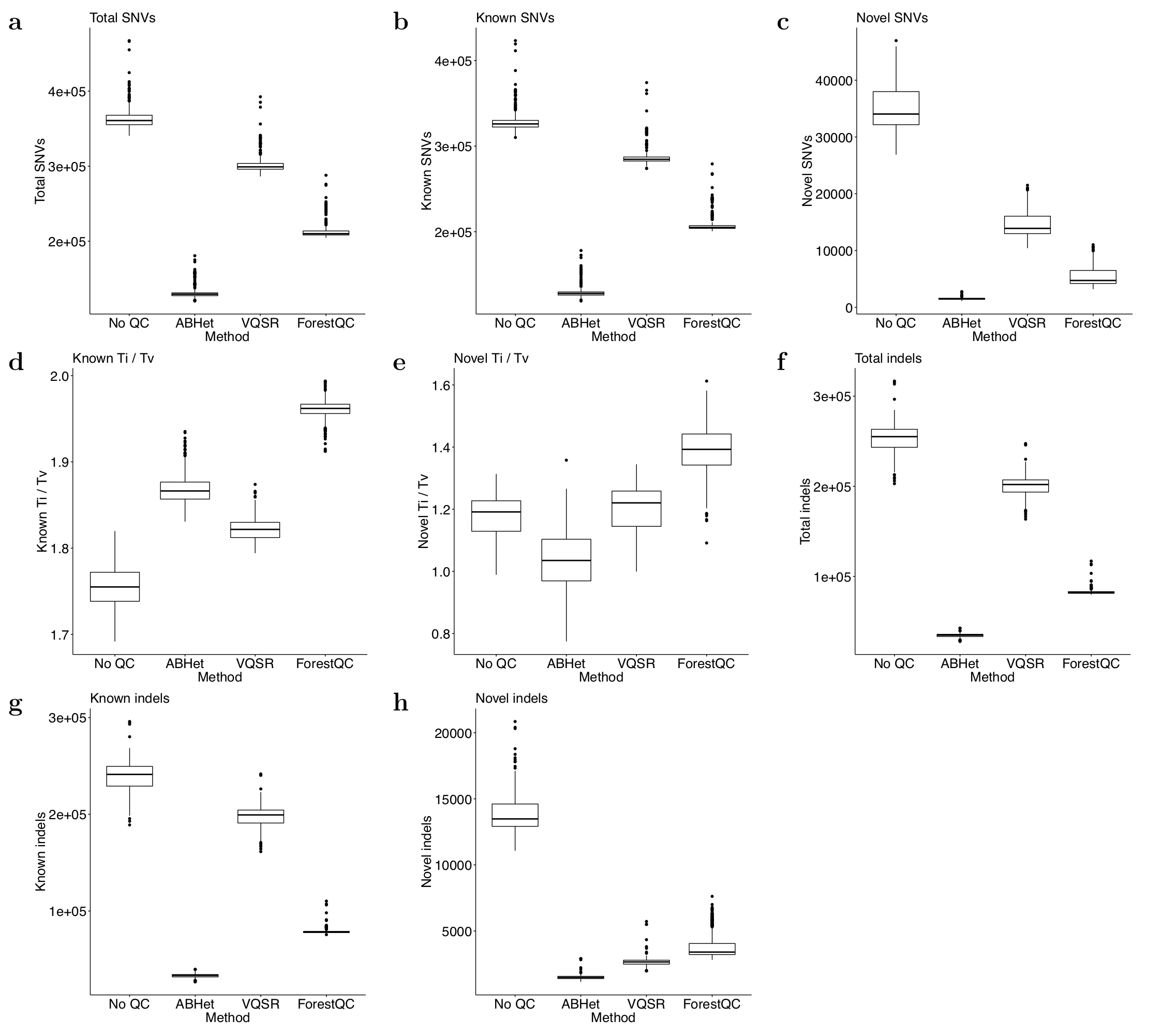

Supplement: S16 Fig — (a) The total number of SNVs. (b) The number of SNVs found in dbSNP. (c) The number of SNVs not found in dbSNP. (d) Ti/Tv ratio of SNVs found in dbSNP. (e) Ti/Tv ratio of SNVs not found in dbSNP. (f) The total number of indels. (g) The number of indels found in dbSNP. (h) The number of indels not found in dbSNP. The version of dbSNP is 150. (TIFF) [file pcbi.1007556.s016.tiff]

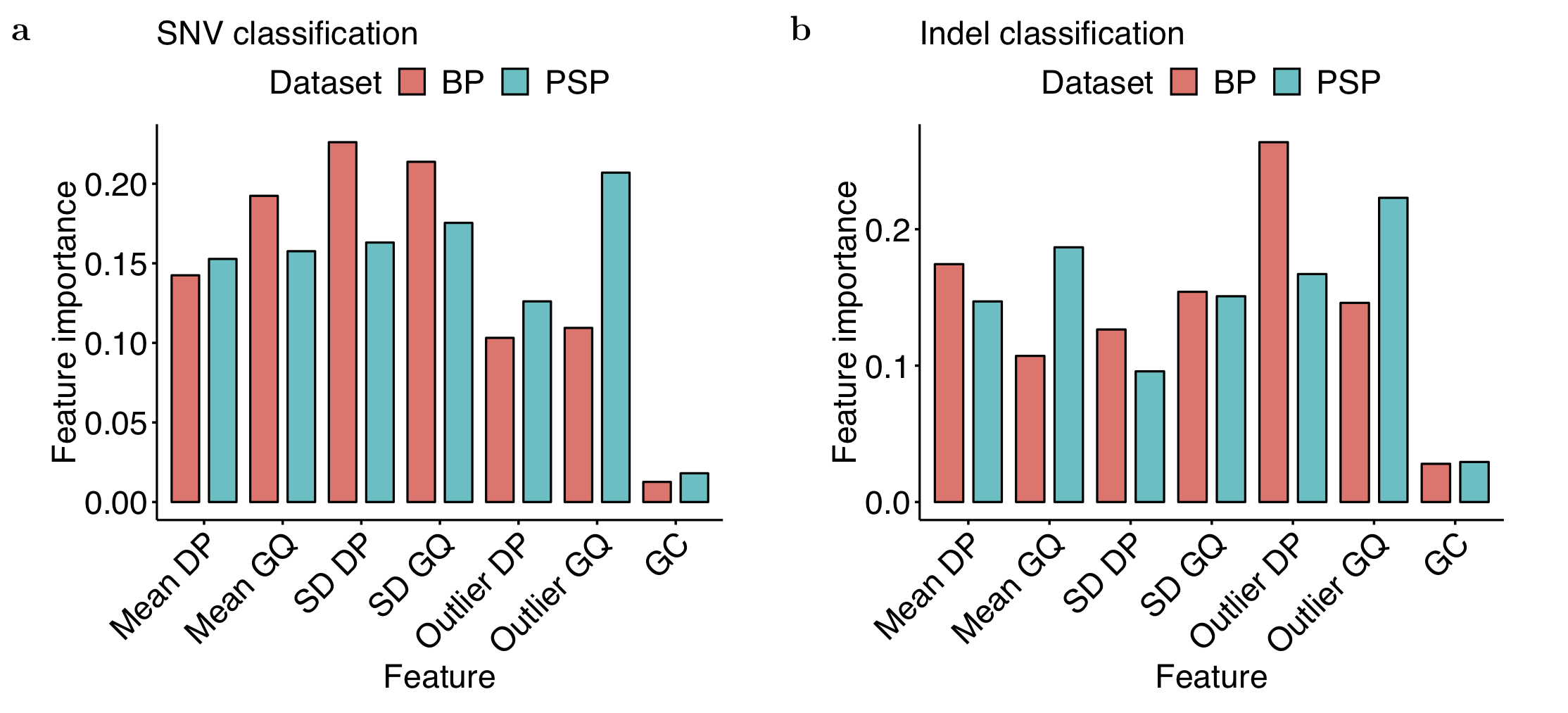

Supplement: S17 Fig — DP stands for sequencing depth. GQ stands for genotyping quality. SD means standard deviation. Outlier DP or GQ means the proportion of samples having genotyping quality or sequencing depth lower than the first quartile of depth or genotyping quality in chromosome 1. GC stands for the GC content of a 1000-bp window where the variant is located. (a) Feature importance in SNV classification. (b) Feature importance in indel classification. (TIFF) [file pcbi.1007556.s017.tiff]

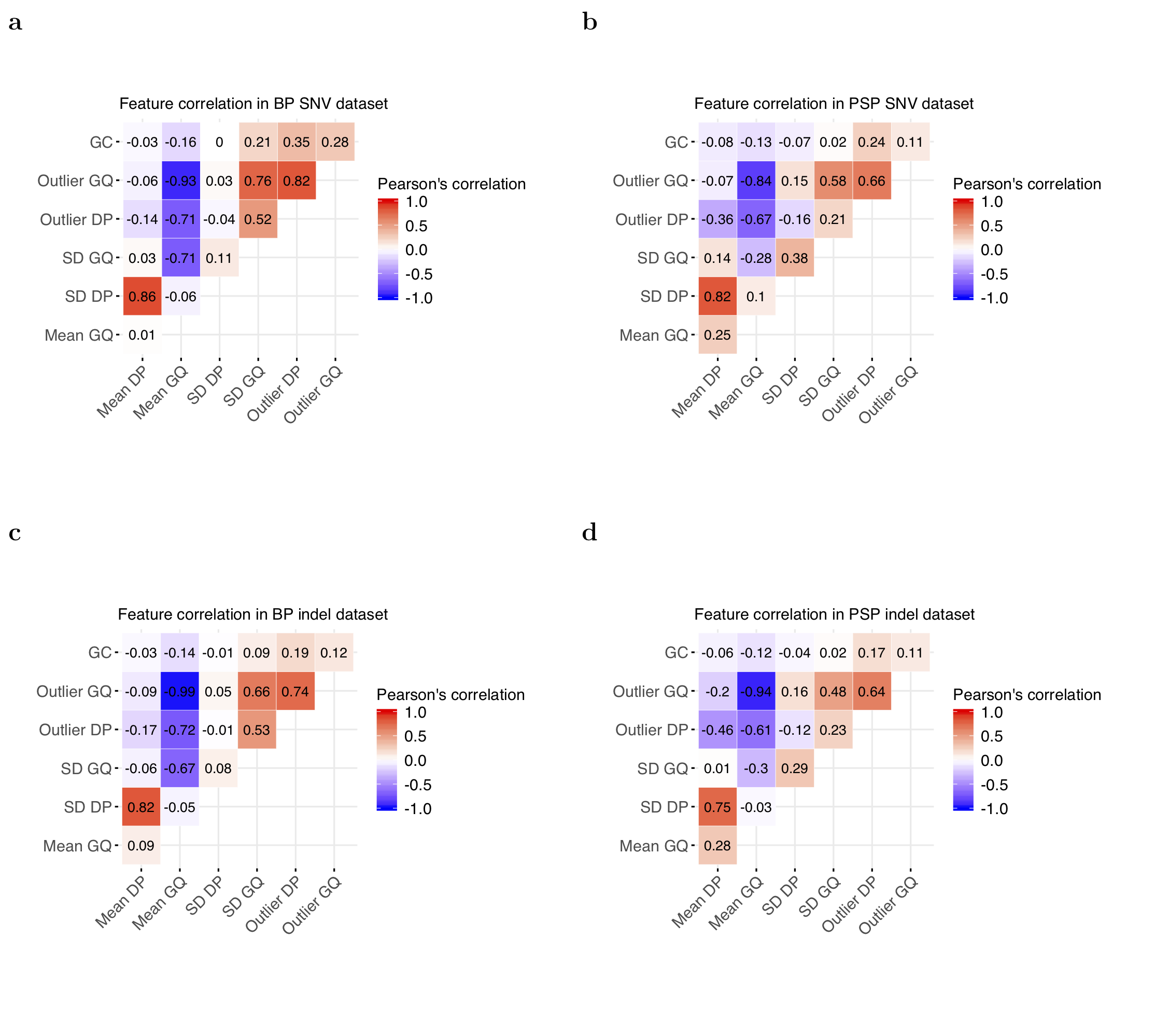

Supplement: S18 Fig — : Pearson’s correlation coefficients between each pair of features in (a) the BP SNV dataset, (b) the PSP SNV dataset, (c) the BP indel dataset, and (d) the PSP indel dataset. (TIFF) [file pcbi.1007556.s018.tiff]

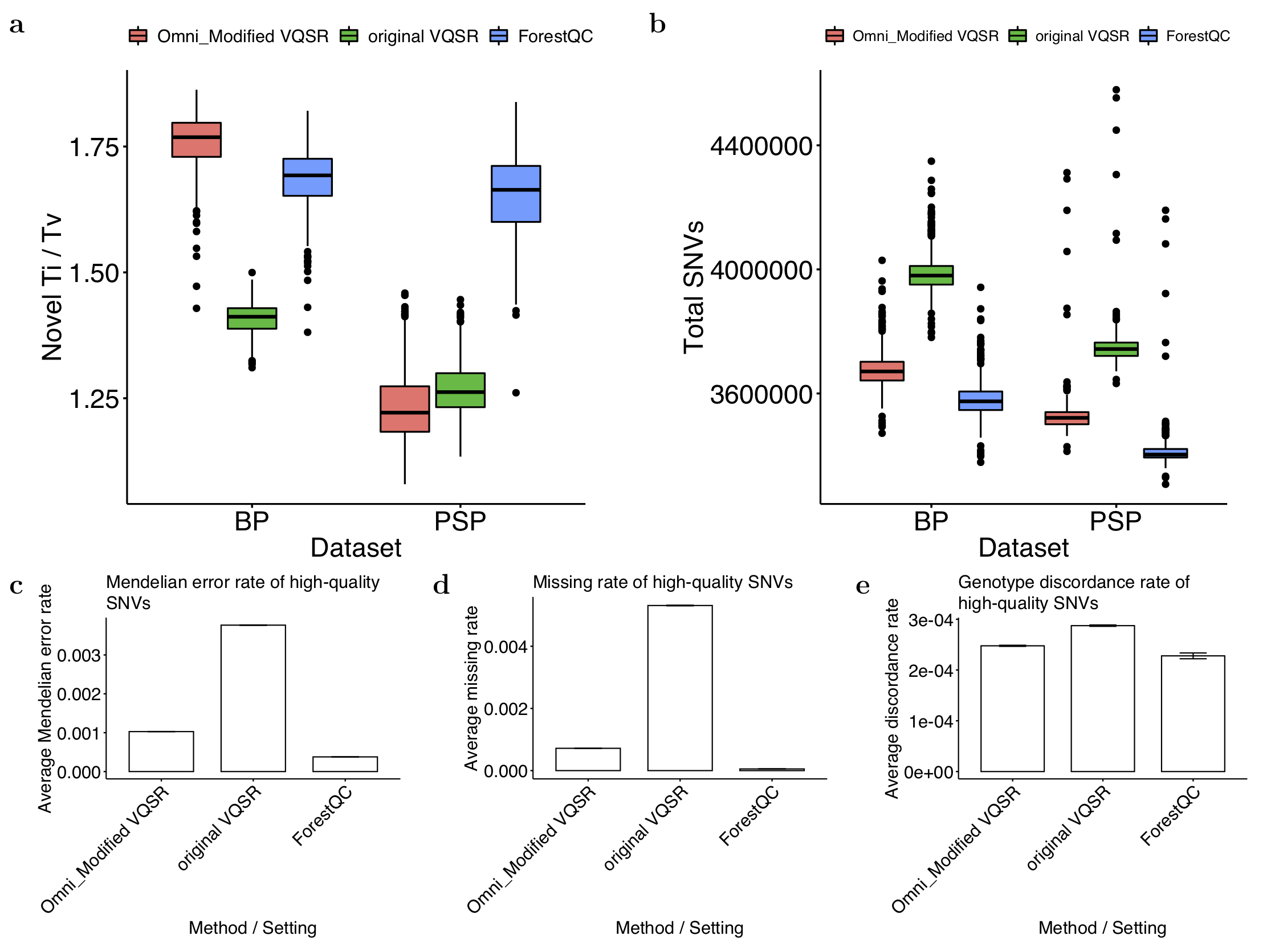

Supplement: S19 Fig — (a) Ti/Tv ratio of SNVs not found in dbSNP v150 and (b) the total number of SNVs in the BP and PSP dataset. (c)-(e) Average Mendelian error rate, average genotype missing rate, and average genotype discordance rate of high-quality SNVs in the BP dataset. Data are represented as the mean ± SEM. “Omni_Modified VQSR”: SNVs in the Omni chip array call set are considered to contain both true- and false-positive sites. “original VQSR”: SNVs in Omni chip array call set are considered to contain only true sites. (TIFF) [file pcbi.1007556.s019.tiff]
