# Supplementary material for: ForestQC: Quality control on genetic variants from next-generation sequencing data using random forest
Source: PLoS Comput Biol. 2019 Dec 18;15(12):e1007556. doi: 10.1371/journal.pcbi.1007556 (PMC6938691; doi:10.1371/journal.pcbi.1007556)
Supplement: S1 Table — (DOCX) [file pcbi.1007556.s020.docx]

**Table S1: Thresholds of four filters for the selection of high-quality variants from the original dataset**

| Filter | Threshold |
| --- | --- |
| Mendelian error rate | 0 |
| Missing rate | < 0.5% |
| HWE p-value | > 0.01 |
| ABHet | 0.3 – 0.7 |

Each high-quality variant must satisfy all thresholds. “HWE p-value”: p-value in testing for Hardy-Weinberg equilibrium.
