# Supplementary material for: ForestQC: Quality control on genetic variants from next-generation sequencing data using random forest
Source: PLoS Comput Biol. 2019 Dec 18;15(12):e1007556. doi: 10.1371/journal.pcbi.1007556 (PMC6938691; doi:10.1371/journal.pcbi.1007556)
Supplement: S2 Table — (DOCX) [file pcbi.1007556.s021.docx]

**Table S2: Thresholds of four filters for the selection of low-quality variants from the original dataset**

| Condition | Filter | Rare variants  (MAF < 0.03) | Common variants  (MAF $\geq$ 0.03) |
| --- | --- | --- | --- |
| ALL | Mendelian error rate | > 3 / (# of trios) | > 5 / (# of trios) |
|  | Missing rate | > 2% | > 3% |
|  | HWE p-value | < 0.005 | < 0.0005 |
|  | ABHet | > 0.75 or < 0.25 | > 0.75 or < 0.25 |
| ANY | Mendelian error rate | > 8 / (# of trios) | > 10 / (# of trios) |
|  | Missing rate | > 8% | > 10% |
|  | HWE p-value | < 0.001 | < 1e-8 |

ALL means all thresholds should be satisfied. ANY means variants are considered low-quality if they satisfy any one of the thresholds. Note that rare variants (MAF < 0.03) and common variants (MAF $\geq$ 0.03) have different thresholds. “HWE p-value”: p-value in testing for Hardy-Weinberg equilibrium.
