# Supplementary material for: ForestQC: Quality control on genetic variants from next-generation sequencing data using random forest
Source: PLoS Comput Biol. 2019 Dec 18;15(12):e1007556. doi: 10.1371/journal.pcbi.1007556 (PMC6938691; doi:10.1371/journal.pcbi.1007556)
Supplement: S3 Table — (DOCX) [file pcbi.1007556.s022.docx]

**Table S3: Accuracy of eight different machine learning algorithms**

| Machine learning algorithm | Accuracy in  SNV classification | Accuracy in  indel classfication |
| --- | --- | --- |
| Random Forest | 0.9736 $\pm$ 0.0018 | 0.9428 $\pm$ 0.0024 |
| ANN | 0.9707 $\pm$ 0.0016 | 0.9401 $\pm$ 0.0027 |
| SVM | 0.9703 $\pm$ 0.0018 | 0.9380 $\pm$ 0.0030 |
| AdaBoost | 0.9671 $\pm$ 0.0016 | 0.9284 $\pm$ 0.0035 |
| Logistic Regression | 0.9666 $\pm$ 0.0010 | 0.9083 $\pm$ 0.0053 |
| KNN | 0.9478 $\pm$ 0.0036 | 0.9197 $\pm$ 0.0038 |
| QDA | 0.9254 $\pm$ 0.0057 | 0.8988 $\pm$ 0.0055 |
| Native Bayes | 0.8973 $\pm$ 0.0073 | 0.8717 $\pm$ 0.0052 |

Accuracy are estimated by performing 10-fold cross-validation. Algorithms are ranked by accuracy in SNV classification. Random forest, ANN, logistic regression and KNN are set to run with eight threads. “ANN”: artificial neural network. “SVM”: single support vector machine. “KNN”: K-nearest neighbors classifier. “QDA”: quadratic discriminant analysis.
