# Supplementary material for: ForestQC: Quality control on genetic variants from next-generation sequencing data using random forest
Source: PLoS Comput Biol. 2019 Dec 18;15(12):e1007556. doi: 10.1371/journal.pcbi.1007556 (PMC6938691; doi:10.1371/journal.pcbi.1007556)
Supplement: S4 Table — (DOCX) [file pcbi.1007556.s023.docx]

**Table S4: Variant-level quality metrics of variants in the BP dataset processed by ForestQC with different settings**

| Metric | ForestQC | ForestQC (ME rate not used) |
| --- | --- | --- |
| Total SNVs | 22227503 | 22301653 |
| Known SNVs | 19361635 | 19401450 |
| Known SNVs (%) | 87.11% | 87.00% |
| Novel SNVs | 2865868 | 2900203 |
| Novel SNVs (%) | 12.89% | 13.00% |
| Known Ti/Tv | 2.1678 | 2.1620 |
| Novel Ti/Tv | 1.7790 | 1.7577 |
| Total indels | 2789037 | 2813369 |
| Known indels | 2237002 | 2251421 |
| Known indels (%) | 80.21% | 80.03% |
| Novel indels | 552035 | 561948 |
| Novel indels (%) | 19.79% | 19.97% |
| Multi-allelic SNVs | 77693 | 78220 |
| Multi-allelic SNVs (%) | 0.35% | 0.35% |
| Known multi-allelic SNVs | 75107 | 75378 |
| Known multi-allelic SNVs (%) | 0.39% | 0.39% |
| Singletons in SNVs | 3801389 | 3804176 |
| Singletons in SNVs (%) | 17.10% | 17.06% |
| Singletons in indels | 433222 | 433035 |
| Singletons in indels (%) | 15.53% | 15.39% |

There are 20 metrics in total, which are described in Material and Methods section in detail. “Known” stands for variants in dbSNP. “Novel” stands for variants not in dbSNP. The version of dbSNP is 150.
