# Supplementary material for: ForestQC: Quality control on genetic variants from next-generation sequencing data using random forest
Source: PLoS Comput Biol. 2019 Dec 18;15(12):e1007556. doi: 10.1371/journal.pcbi.1007556 (PMC6938691; doi:10.1371/journal.pcbi.1007556)
Supplement: S6 Table — (DOCX) [file pcbi.1007556.s025.docx]

**Table S6: Rare variants and common variants in the BP dataset processed by different methods**

| Method | Rare SNVs | Common SNVs | Rare indels | Common indels |
| --- | --- | --- | --- | --- |
| No QC | 16304019 (65.00%) | 8777577  (35.00%) | 2219301 (55.81%) | 1757402 (44.19%) |
| ABHet | 14682507 (65.50%) | 7732821  (34.50%) | 1655360 (61.98%) | 1015280 (38.02%) |
| VQSR | 15908575 (65.63%) | 8330751  (34.37%) | 1667942 (51.91%) | 1544939 (48.09%) |
| ForestQC | 14952779 (67.27%) | 7274684  (32.73%) | 1876432 (67.28%) | 912598 (32.72%) |

The number and fraction of rare variants (MAF < 0.03) and common variants (MAF $\geq$ 0.03) in all high-quality variants identified by different methods in the BP dataset.
