# Supplementary material for: ForestQC: Quality control on genetic variants from next-generation sequencing data using random forest
Source: PLoS Comput Biol. 2019 Dec 18;15(12):e1007556. doi: 10.1371/journal.pcbi.1007556 (PMC6938691; doi:10.1371/journal.pcbi.1007556)
Supplement: S8 Table — (DOCX) [file pcbi.1007556.s027.docx]

**Table S8: Variant-level quality metrics of high-quality variants in the PSP dataset processed by four different methods**

| Metric | No QC | ABHet | VQSR | ForestQC |
| --- | --- | --- | --- | --- |
| Total SNVs | 33273111 | 29771182 | 31281620 | 29352329 |
| Known SNVs | 25960464 | 24142744 | 24910728 | 23514257 |
| Known SNVs (%) | 78.02% | 81.09% | 79.63% | 80.11% |
| Novel SNVs | 7312647 | 5628438 | 6370892 | 5838072 |
| Novel SNVs (%) | 21.98% | 18.91% | 20.37% | 19.89% |
| Known Ti / Tv | 2.1202 | 2.1811 | 2.1643 | 2.1990 |
| Novel Ti / Tv | 1.6253 | 1.8236 | 1.7089 | 1.8131 |
| Total indels | 5093443 | 3311136 | 3682319 | 3418242 |
| Known indels | 3679990 | 2532899 | 3012662 | 2567879 |
| Known indels (%) | 72.25% | 76.50% | 81.81% | 75.12% |
| Novel indels | 1413453 | 778237 | 669657 | 850363 |
| Novel indels (%) | 27.75% | 23.50% | 18.19% | 24.88% |
| Multiallelic SNVs | 250418 | 6685 | 188180 | 146247 |
| Multiallelic SNVs (%) | 0.75% | 0.02% | 0.60% | 0.50% |
| Known multiallelic SNVs | 219411 | 6210 | 174818 | 138890 |
| Known multiallelic SNVs (%) | 0.85% | 0.03% | 0.70% | 0.59% |
| Singletons in SNVs | 14768613 | 13849361 | 14390515 | 14055519 |
| Singletons in SNVs (%) | 44.39% | 46.52% | 46.00% | 47.89% |
| Singletons in indels | 1582090 | 1350433 | 1242934 | 1393736 |
| Singletons in indels (%) | 31.06% | 40.78% | 33.75% | 40.77% |

Four methods are compared, including no QC applied, ABHet approach, VQSR and ForestQC. There are 20 metrics in total, which are described in Material and Methods section in detail. “Known” stands for variants found in dbSNP. “Novel” stands for variants not found in dbSNP. The version of dbSNP is 150.
