# Supplementary material for: ForestQC: Quality control on genetic variants from next-generation sequencing data using random forest
Source: PLoS Comput Biol. 2019 Dec 18;15(12):e1007556. doi: 10.1371/journal.pcbi.1007556 (PMC6938691; doi:10.1371/journal.pcbi.1007556)
Supplement: S9 Table — (DOCX) [file pcbi.1007556.s028.docx]

**Table S9: Rare variants and common variants in the PSP dataset processed by different methods**

| Method | Rare SNVs | Common SNVs | Rare indels | Common indels |
| --- | --- | --- | --- | --- |
| No QC | 24864011 (74.73%) | 8409100  (25.27%) | 3381339  (66.39%) | 1712104 (33.61%) |
| ABHet | 22005560 (75.04%) | 7321250 (24.96%) | 2337310  (72.66%) | 879481 (27.34%) |
| VQSR | 23593775 (75.42%) | 7687845 (24.58%) | 2349383  (63.80%) | 1332936 (36.20%) |
| ForestQC | 22525090 (76.74%) | 6827239 (23.26%) | 2603084  (76.15%) | 815158 (23.85%) |

The number and fraction of rare variants (MAF < 0.03) and common variants (MAF $\geq$ 0.03) in all high-quality variants identified by different methods in PSP dataset.
