# Supplementary material for: ForestQC: Quality control on genetic variants from next-generation sequencing data using random forest
Source: PLoS Comput Biol. 2019 Dec 18;15(12):e1007556. doi: 10.1371/journal.pcbi.1007556 (PMC6938691; doi:10.1371/journal.pcbi.1007556)
Supplement: S11 Table — (DOCX) [file pcbi.1007556.s030.docx]

**Table S11: Running time of ForestQC and VQSR in two datasets, measured in real time**

| Method | BP SNV | BP indel | PSP SNV | PSP indel |
| --- | --- | --- | --- | --- |
| ForestQC | 17.00 min | 3.74 min | 23.24 min | 5.82 min |
| VQSR | 6.03 h | 1.21 h | 8.30 h | 1.44 h |
