# Supplementary material for: ForestQC: Quality control on genetic variants from next-generation sequencing data using random forest
Source: PLoS Comput Biol. 2019 Dec 18;15(12):e1007556. doi: 10.1371/journal.pcbi.1007556 (PMC6938691; doi:10.1371/journal.pcbi.1007556)
Supplement: S12 Table — (DOCX) [file pcbi.1007556.s031.docx]

**Table S12: Definitions of 23 metrics for sequencing quality control calculated for sample-level and variant-level**

| Metric | Definition | Sample-level  or Variant-level |
| --- | --- | --- |
| Het / Hom | (# of heterozygous calls) / (# of homozygous non-reference calls) | Sample-level only |
| Total SNVs | The total number of SNVs | Both |
| Total SNVs (%) | The proportion of the total SNVs of a sample in the total SNVs of the entire dataset | Sample-level only |
| Known SNVs | The number of SNVs found in dbSNP | Both |
| Known SNVs (%) | The proportion of SNVs found in dbSNP | Both |
| Novel SNVs | The number of SNVs not found in dbSNP | Both |
| Novel SNVs (%) | The proportion of SNVs not found in dbSNP | Both |
| Known Ti/Tv | The Ti/Tv ratio of the known SNVs | Both |
| Novel Ti/Tv | The Ti/Tv ratio of the novel SNVs | Both |
| Total indels | The total number of indels | Both |
| Total indels (%) | The proportion of the total indels of a sample in the total indels of the entire dataset | Sample-level only |
| Known indels | The number of indels found in dbSNP | Both |
| Known indels (%) | The proportion of indels found in dbSNP | Both |
| Novel indels | The number of indels not found in dbSNP | Both |
| Novel indels (%) | The proportion of indels not found in dbSNP | Both |
| Multi-allelic SNVs | The number of multi-allelic SNVs | Variant-level only |
| Multi-allelic SNVs (%) | The proportion of multi-allelic SNVs | Variant-level only |
| Known multi-allelic SNVs | The number of multi-allelic SNVs found in dbSNP | Both |
| Known multi-allelic SNVs (%) | The proportion of multi-allelic SNVs found in dbSNP | Both |
| Singletons in SNVs | The number of singletons in SNVs | Both |
| Singletons in SNVs (%) | The proportion of singletons in SNVs | Both |
| Singletons in indels | The number of singletons in indels | Both |
| Singletons in indels (%) | The proportion of singletons in indels | Both |

Only three metrics, (Het / Hom, % Total SNVs and % total indels) are only calculated for sample-level. Other metrics are measured for every variant site and every sample. The version of dbSNP used in this study is 150.
